# A review of recent advances in anesthetic drugs for patients undergoing cardiac surgery

**DOI:** 10.3389/fphar.2025.1533162

**Published:** 2025-02-18

**Authors:** Yutian Sun, Xiangyou Sun, Haibo Wu, Zhaoyang Xiao, Wei Luo

**Affiliations:** ^1^ Department of Cardiac Surgery, The Second Hospital of Dalian Medical University, Dalian, Liaoning, China; ^2^ Department of Anesthesiology, The Second Hospital of Dalian Medical University, Dalian, Liaoning, China

**Keywords:** cardiovascular anesthesia, cardiovascular surgery, new anesthetic drugs, drug delivery systems, cardiac surgery

## Abstract

Inducing and maintaining general anesthesia requires a diverse set of medications. Currently, heart surgery anesthetic management does not adhere to any one standard protocol or set of drugs. To ensure steady circulatory function while providing sufficient sedation, anesthetic medications are carefully selected for cardiovascular operations. Among the opioids used most often in cardiac surgery are fentanyl, sufentanil, and remifentanil. As a cardiac anesthesiologist, your key responsibilities will be to maintain your patient’s blood pressure (BP) and oxygen levels, reduce the frequency and intensity of ischemia events, and make it easy for them to get off of cardiopulmonary bypass (CPB) and supplemental oxygen fast. Additionally, new knowledge gaps have been identified as a result of developments in cardiac anesthetics, which must be addressed. The goal of the most recent developments in cardiac anesthesia has been to decrease risks and increase accuracy in patient outcomes during cardiac surgeries. Furthermore, new methods and tools are contributing to the evolution of cardiovascular anesthesia toward a more dynamic, patient-centered approach, with an eye on boosting safety, decreasing complications, and facilitating better recovery for patients. New medications and methods have emerged in the field of anesthetic pharmacology, aiming to improve anesthesia management, particularly for patients who have cardiovascular disease. Optimal cardiovascular stability, fewer side effects, and enhanced surgical recovery are achieved by use of these medications. We have reviewed all the different kinds of cardiac anesthetic techniques and medications in this research. We have also examined the many new anesthetic medicines that have been produced and used for individuals with cardiovascular issues. Next, we covered prospects in the realm of cardiovascular anesthesia and novel cardiac anesthetic drugs.

## Graphical Abstract

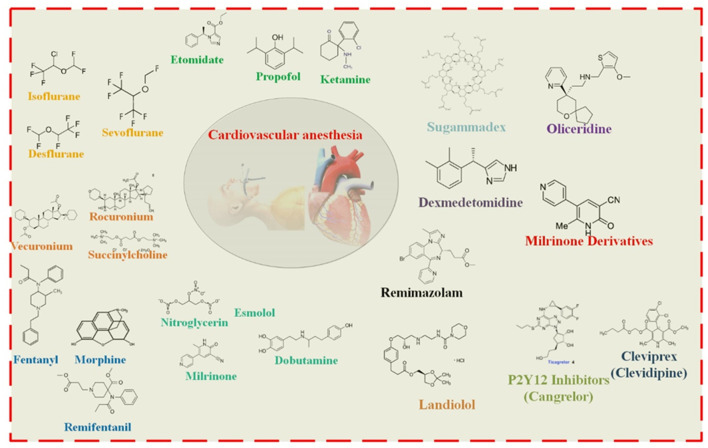

## 1 Introduction

Research into the mechanisms of action of general anesthetics is a continuous endeavor. Finding ways to reverse general anesthesia has been the focus of a separate area of study. Patients may have a faster recovery and fewer postoperative problems if reversal drugs are used. The search for medicines that can reverse the effects of general anesthesia has been hindered by our current lack of understanding of its underlying mechanisms. Nevertheless, efforts to develop reversal drugs have enhanced our knowledge of these processes. Research into possible reversal medicines has shown how critical it is to use strict criteria when testing animals for recovery from general anesthesia. Essential systems of arousal (such as the cholinergic, dopaminergic, and orexinergic systems) that are important for awakening from general anesthesia have been identified with its aid. In addition, several general anesthetics show varied effects when administered with reversal agents, suggesting that their underlying processes are distinct. Recovery from general anesthesia likely involves not only postsynaptic receptors but also the presynapse and glia. Alternative processes involving the tripartite synapse will need to be considered in the subsequent phase of the reversal agent search (Cylinder et al., 2024).

Cardiac surgery anesthesia is intricate, requiring the careful balancing of general anesthetic’s fundamental goals with the need to maintain hemodynamic stability, protect organs, and preserve myocardial function—all while dealing with the unique challenges of cardiopulmonary bypass (CPB). Achieving this involves the use of a wide variety of anesthetic agents. Nevertheless, there is still no conclusive evidence supporting the superiority of any specific agent or method. The potential myocardial-protecting characteristics of volatile anesthetics and the preference to avoid modern long-acting intravenous drugs have sustained their popularity during CPB since their introduction in 1974. Patients with a low circulatory reserve were first identified as benefiting from high-dose opioid administration during cardiac anesthesia in 1969, and newer ultra-short-acting opioids are being used today. In the early 2000s, propofol was introduced as a short-acting intravenous alternative to volatile agents and high-dose opioids, providing clinicians with another viable option (O’Gara et al., 2023).

The idea of opioid-free anesthesia (OFA) has been around for a while. It is based on the observation that hemodynamic changes indicating a sympathetic response in a patient under anesthesia do not consistently represent pain. Furthermore, when a patient is asleep, they will not remember any pain, and other therapeutic classes to opioids may reduce hormonal stress and sympathetic and inflammatory responses. A growing body of research on OFA demonstrates its feasibility, resulting in better postoperative health and less need for morphine. Additionally, other OFA protocols have been published. There has been individual research on lidocaine, dexamethasone, and ketamine, the three most popular non-opioid analgesics used in cardiac surgery. The administration of dexamethasone reduced morphine consumption and duration of stay in the intensive care unit (ICU), as shown by Murphy et al. Researchers indicated that ketamine may alleviate pain and reduce the need for opioids. Both cardiac and non-cardiac surgeries have demonstrated that lidocaine can effectively reduce pain and minimize the need for opioids (Peltoniemi et al., 2016; Mauermann et al., 2017; Murphy et al., 2011; Guinot et al., 2019).

Minimally invasive techniques are gradually replacing the classic sternotomy in heart surgery. Under Dr. Joseph T. McGinn’s direction, a team of surgeons in New York City conducted the first minimally invasive heart surgery in 2005. Anesthesiologists are essential to obtain the best possible results from these types of treatments. The anesthesia staff has unique problems while managing these patients after surgery. Regional anesthesia, analgesia, and thoracic anesthesia (specifically, one-lung ventilation (OLV), cardiac anesthesia, and transesophageal echocardiography (TEE)) are just a few of the many subspecialties that anesthesiologists need to be proficient in (Balasubramanyam and Kapoor, 2019). Worldwide, surgical specialties are quickly adopting Enhanced Recovery After Surgery (ERAS) as a perioperative patient care technique. All parts of ERAS work together throughout the perioperative period to help patients and the healthcare system as a whole. While ERAS in cardiac surgery (ERAS-C) has the potential to bring about comparable advancements, there is a lack of confusion around the usage of ERAS-C programs at present. Preliminary findings from ERAS-C trials have demonstrated advantages similar to those observed in other areas of surgery. These include shorter hospital stays (1–4 days) and shorter times spent in the ICU (4–20 h), better management of pain during surgery (25%–60% less opioid use), and better outcomes in early postoperative mobility and oral diets. Beneficial outcomes for cardiac surgery have also been shown, with a reported 8%–14% reduction in the occurrence of postoperative atrial fibrillation (POAF) (Baxter et al., 2020).

The use of drug delivery systems (DDSs) to alter the pharmacokinetic characteristics of already-existing pharmaceuticals has been the center of recent pharmacological studies in local anesthetics (LAs). The most glaring example of this is liposomal bupivacaine (LB), which has a longer half-life and a respectable safety record. Nevertheless, several studies conducted since its launch in 2011 have shown contradictory findings regarding its effectiveness, casting doubt on the merit of its value proposition. In addition, compared to LB alone, trials investigating the efficacy of adjuvant drugs such as epinephrine, dexamethasone, and dexmedetomidine (DEX) have shown encouraging outcomes. New LA DDSs include bupivacaine implants for hernia repairs and extended-release bupivacaine for arthroscopy-related local infiltration. So far, both products have shown intriguing but limited outcomes. More studies are required to determine the effectiveness of these products. Due to the current clinical studies showing a flood of new LAs being introduced on the market with unique DDSs, this study will play a crucial role in the near future (Syed et al., 2024).

In this study, we reviewed the many cardiac anesthetic medications available and some of the more recent innovations in this field. Future cardiovascular anesthetic research in the fields of pharmacology, personalized medicine, and technological integration aims to improve patient safety, enhance results, and allow speedier recovery. So we examined these novel anesthetic techniques in heart patients in our research.

## 2 Common anesthetic drugs in cardiovascular patients

Analgesia, amnesia, numbness, and muscle relaxation are all symptoms of general anesthesia, which is a type of full-body numbness. A wide range of substances can be used to achieve general anesthesia, either partly or entirely. There is currently no agreed-upon method for administering anesthesia during heart surgery. Instead, the anesthesiologist’s personal choice and the patient’s pathophysiologic condition determine the substances and medication combinations employed. Hypnosis, analgesia, amnesia, and muscular relaxation are the four critical components of modern procedures that make up general anesthesia, as defined. It seems to reason that medications with multiple effects are often administered together to maximize their efficacy, although many of the compounds discussed in this study are capable of having more than one impact (Alwardt et al., 2005).

When a patient is put into a state of controlled unconsciousness under general anesthesia, medical procedures may be carried out more effectively. Historically, hypnosis, analgesia, and muscular relaxation have been considered the three main goals of anesthesia. First, the patient is evaluated before the operation starts. Then, all necessary supplies and medications are double-checked to make sure they are safe to use. The induction of anesthesia begins with the administration of induction drugs, either intravenously or via inhalation. During the induction period of anesthesia, it is crucial to think about airway care. Many factors, such as the patient’s age, physiology, type of operation, co-morbidities, and other medical conditions, influence the selection and dosage of the induction agent. Both inhalation and intravenous administration are acceptable methods for administering the maintenance phase of anesthesia after induction. Quick reduction of airway reflexes and alterations in cardiorespiratory function are common side effects of anesthetic medication delivery. Consequences of general anesthesia include heightened consciousness, aspiration, anaphylaxis, airway narrowing, laryngospasm, unstable cardiovascular system, abnormally high body temperature, and harm to the teeth and gums. To reduce the likelihood of these problems, careful attention to detail is required (Scott et al., 2024).

Interventional cardiology is a rapidly evolving field. Cardiovascular diseases (CVDs) that were once treated with heart surgery are now managed with several complicated treatments performed outside the operating room. Improving patient comfort and achieving a successful surgery while guaranteeing safety necessitate an appropriate sedation strategy. Sedation for cardiovascular intervention might be particularly challenging in very sick, high-risk patients. With the right kind of sedation, patients may remain completely still during the process, which increases the likelihood of a good outcome, decreases the possibility of complications due to environmental optimization, and boosts patient happiness. Mainly, when dealing with critically ill, high-risk patients having complicated operations, it might be challenging to accomplish this aim. To ensure safe and effective sedation during cardiovascular procedures, clinicians must possess a thorough understanding of sedative and analgesic pharmacology, have good monitoring skills, and be able to rescue patients from profound sedation or other associated complications (Song et al., 2020).

Researchers examined the effects of etomidate (ETO), midazolam (DMZ), and DEX on the cardiovascular system during phacoemulsification procedures using local topical anesthesia. The subjects of this randomized, double-blind clinical experiment were 90 individuals receiving phacoemulsification as a candidate for cataract surgery. The first group was given 1 μg/kg of DEX for 10 min, after which they were given an infusion of the drug at a rate of 0.5 μg/kg/h. One group was given a gradual intravenous injection of 0.2 mg/kg of DMZ, whereas the other two groups were given 0.05 mg/kg of the drug. All patients had their vitals monitored before, during, and after anesthesia, as well as their sedation levels and any side effects. This research found that DEX, DMZ, and ETO all produced comparable levels of sedation. Researchers suggested that in terms of maintaining stable BP and pulse rate, ETO seemed to be more effective than the other two medications (Shoraibi et al., 2024).

### 2.1 Etomidate in patients undergoing cardiac surgery

The induction of general anesthesia and sedation is accomplished with the use of ETO, an imidazole-based agonist of the γ-aminobutyric acid type A (GABAA) receptor. Much like barbiturates and propofol, ETO acts primarily on the GABAA receptor to provide a rapid hypnotic onset. It works by boosting the effects of the inhibitory neurotransmitter γ-aminobutyric acid by acting as a positive allosteric modulator on the GABAA receptor. Unlike other anesthetics, ETO does not cause any depression in the circulatory or respiratory systems, making it stand out with its very stable cardiorespiratory profile. Nevertheless, ETO inhibits the 11β-hydroxylase enzyme, which, in turn, suppresses the adrenocortical axis. Because of this, the medicine should not be given by an extended infusion. Additionally, this renders the medication inappropriate for use in patients who are in a severe condition. ETO is hydrolyzed by hepatic esterases into an inert carboxylic acid and has a reasonably high volume of distribution. Due to its declining popularity, there are not many comprehensive pharmacokinetic or pharmacodynamic investigations available today (Valk and Struys, 2021).

The favorable circulatory profile of ETO makes it a preferred choice for inducing anesthesia in patients undergoing heart surgery. The GABA receptor complexes in the central nervous system (CNS) are responsible for the effects of hypnotic agents. The therapeutic efficacy of ETO and other medications has been the subject of several comparison studies. However, there is still no clear consensus on the relative safety and effectiveness of ETO. Researchers conducted a comprehensive review of randomized controlled trials to determine the effect of ETO on cardiac surgery patients in terms of adverse events and patient outcomes. After induction and intubation, patients who received ETO had a more stable hemodynamic profile than those who received comparator medications. After induction and intubation, patients treated with ETO were far less likely to need a vasopressor than those treated with comparator medications. The included studies showed a considerable amount of heterogeneity in this comprehensive meta-analysis. Furthermore, the hemodynamic profile of ETO was the only focus of the majority of the research. Therefore, this setting is not appropriate for answering questions about the safety and effectiveness of ETO. However, compared to other medications used during induction and intubation, ETO seems to have limited cardiovascular benefits for individuals having cardiac surgery. Since ETO did not affect mortality, tracheal intubation time, ICU stay, or hospital length of stay (LOS), improved hemodynamics did not lead to better clinical outcomes. In conclusion, data were generally absent, making it impossible to examine infectious adverse effects as a significant factor contributing to increased mortality in ICU patients owing to ETO administration (Xia et al., 2024).

ETO is a non-barbiturate sedative with an ultra-short half-life and potent inhibitory effects on the CNS. Its most typical use is to induce aided or general anesthesia via intravenous injection. Recently, ETO has emerged as a substitute for opioids and new psychoactive substances, leading to an increase in abuse. Chronic ETO overdose can result in permanent brain damage and other mental health issues. Cognitive impairments, behavioral problems, self-mutilation, and mortality might be symptoms of a severe case. Little is known about the toxicological processes of ETO. Furthermore, reliable procedures for detecting ETO in blood, urine, and hair are not yet available, and there is a dearth of knowledge about the clinical signs and histological changes linked to ETO poisoning. Therefore, it is essential to conduct more studies on toxicological pathology and establish trustworthy testing procedures (Zheng et al., 2024).

Furthermore, ETO has a lower incidence of adverse cardiac events than propofol while offering sedative effectiveness that is comparable or superior to propofol. However, ETO has serious side effects, including pretreatment-needed myoclonus, adrenocortical suppression, and unsubstantiated fatalities associated with it. There has been a shortage of scholarly work and analytical tools pertaining to ETO usage and abuse, although this problem has just recently surfaced in the forensics community. Continuously studying the potential for abuse of ETO and closely monitoring relevant instances are essential for the successful management of its overuse or abuse (Uhm et al., 2024).

Some people misuse ETO, despite its usefulness as a surgical anesthetic. The mechanisms by which ETO impacts the CNS via the gut–brain axis and its long-term effects on brain and intestine functions remain unclear. Researchers examined the gastrointestinal and neurotoxic effects of ETO in mice treated with dosages of 1 mg/kg and 3 mg/kg daily for 14 consecutive days. Researchers’ findings demonstrated that mice developed drug resistance after receiving long-term injections of ETO, which altered their intrinsic dark preference and may have caused dependency. There was a 38% decrease in GABA levels, a 52% decrease in serum levels, and a 42% decrease in 5-hydroxytryptamine levels in the brain, serum, and colon, respectively. Staining with hematoxylin and eosin (H&E) showed that ETO had a negative effect on the intestinal barrier and decreased the number of goblet cells in the colon. Claudin-4 and ZO-1, two genes involved in tight junctions, had their expression downregulated. There was a shift in the gut flora, marked by an 18% increase in *Klebsiella* and decreases of 33% and 14% in *Akkermansia* and *Lactobacillus*, respectively. TUNEL analysis revealed that brain apoptotic cell counts were elevated in response to high-dose ETO. Claudin-1 expression was reduced in the brain. Colon and brain untargeted metabolomics studies linked ETO to glycerophospholipid metabolism disorders. Intestinal flora and metabolism were altered by ETO, which had further effects on the CNS via the gut–brain axis; abnormal lipid metabolism may result in the formation or buildup of lipotoxic compounds, which cause illnesses in the CNS. Research has highlighted the adverse effects of long-term ETO use on the brain and gastrointestinal system, which may help us understand the harmful impacts of ETO misuse on human health (Ding et al., 2024).

ETO, a synthetic imidazole anesthetic, produces hypnotic effects by either directly activating the anionic GABA (GABA-A) receptor or amplifying the action of the inhibitory neurotransmitter GABA. Among anesthetics, it stands out due to its various benefits, including low restriction of spontaneous breathing and high hemodynamic stability. This medication has limited therapeutic utility due to its poor water solubility and adverse effects, such as myoclonus and adrenal cortex inhibition. In response to these concerns, many ETO formulations have emerged as a result of decades of study into the drug delivery of ETO. There have been several ETO formulations; however, not all harmful side effects have been adequately addressed (Zhang et al., 2024a).

### 2.2 Propofol in patients undergoing cardiac surgery

Several investigations have shown that propofol’s anti-inflammatory properties, including a decrease in the production of inflammatory mediators, have cardioprotective benefits. Recall that propofol has several anti-inflammatory benefits when administered during open-heart surgery, particularly before aortic cross-clamp release in patients having elective coronary artery bypass grafting (CABG) surgery. This means that it reduces the effect of lipid peroxides on the myocardium, significantly lessens the likelihood of an inflammatory reaction as a strong reaction to myocardial reperfusion, and limits the inflammatory cascade (Elgebaly et al., 2020; Samir et al., 2015; Corcoran et al., 2004).

Researchers looked at how cardiovascular and cerebrovascular controls were affected by general propofol-based anesthesia. By calculating the cross-correlation function (CCF) between heart period (HP) and systolic arterial pressure (SAP) and between mean arterial pressure (MAP) and mean cerebral blood flow (MCBF) velocity, the latency and strength of cerebrovascular and cardiovascular controls were examined. Twenty-five patients having coronary artery bypass grafting (CABG) (63.4 ± 8.7 years, two female subjects) had their HP, SAP, MAP, and MCBF velocities measured both before and after the introduction of propofol general anesthesia. While subjects were mechanically ventilated during post-exposure surgery (POST), they breathed normally during pre-exposure (PRE). Physiological assumptions on the sign and latency of the assumed dynamic interactions informed the sampling of CCFs at the first peak or valley, with negative and positive delays approaching 0. Associating signal pairings from many participants allowed researchers to test the null hypothesis of uncoupling. The presence of bidirectional connections suggests that closed-loop regulatory mechanisms are involved in HP–SAP regulation through the baroreflex and mechanical feedforward pathways and in MCBF-MAP control via the pressure-to-flow link and the Cushing reflex. Researchers indicated that while propofol anesthesia considerably reduced the intensity of the Cushing reflex, it had no effect on the coupling strength along the baroreflex, feedforward mechanical route, or pressure-to-flow connection. Additionally, the baroreflex and pressure-to-flow link latencies were significantly reduced during propofol anesthesia. As a consequence of changes in autonomic function caused by propofol, the suggested study implies that general anesthesia causes significant alterations to cardiovascular and cerebrovascular regulation (Bari et al., 2021). Furthermore, researchers indicated that compared to propofol, the volatile anesthetic class showed better long-term mortality and other secondary outcomes, suggesting myocardial protection in people having cardiac surgery with CPB (Bonanni et al., 2020).

To evaluate propofol's impact on post-cardiac surgery neurocognitive function after cardiac surgery in comparison to volatile anesthetics, the postoperative neurocognitive function score was the primary endpoint. In contrast, delirium incidence after heart surgery was the secondary result. Neurocognitive function ratings after surgery did not significantly alter from pre- to post-op. Neither the propofol group nor the group using volatile anesthetics had substantially higher rates of delirium. When it comes to postoperative neurocognitive impairment after heart surgery, volatile anesthetics and propofol are comparable, unlike in non-cardiac surgery (Ju et al., 2024).

To minimize the risk of myocardial ischemia, dysfunction, and heart failure after cardiac surgery, it is essential to employ multimodal strategies during the perioperative period to protect the heart from the increased oxygen demand and consumption that occurs during the procedure. In CPB procedures, propofol–DEX is more effective than propofol alone in preventing cardiac complications (Elgebaly et al., 2020).

CABG surgery is an essential method for treating coronary heart disease. The risk of perioperative myocardial injury (PMI) and poor clinical outcomes are increased in patients who undergo this treatment due to advanced age, comorbidities, poor cardiac function, and other medical conditions. Hence, it is essential to continue researching methods to safeguard the delicate heart during CABG operation. Animal studies have shown that volatile anesthetics such as desflurane, isoflurane, and sevoflurane protect cardiac tissue against acute ischemia-reperfusion injury (IRI) by decreasing the size of infarcts. There is still debate among researchers over whether volatile anesthetics have a cardioprotective impact on cardiac troponins during off-pump coronary artery bypass grafting (OPCAB) procedures. Researchers have examined the systematic effects of propofol and volatile anesthetics in patients undergoing OPCAB surgery. Researchers have shown that myocardial damage biomarkers are significantly lower in individuals who received volatile anesthetic compared to those who received propofol. Postoperative cardiac troponin levels were lower in sevoflurane-treated patients than in propofol-treated patients, according to subgroup analysis. Desflurane and isoflurane are alternatives to propofol; however, they do not provide any significant benefits at this time. Time on mechanical breathing after surgery, duration in the ICU, and death rates were not significantly different between the two groups. In adults undergoing OPCAB surgery, researchers found that sevoflurane, a volatile anesthetic, provided greater cardioprotective benefits than propofol (Zhang et al., 2023).

Researchers systematically assessed the literature on postoperative sedation following open heart surgery in adults with DEX or propofol. Researchers showed that DEX may have saved time in the ICU compared to propofol. Still, it did not seem to help patients undergoing heart valve surgery or CABG achieve better outcomes after surgery (Abowali et al., 2021).

### 2.3 Ketamine in patients undergoing cardiac surgery

The N-methyl-d aspartic acid (NMDA) antagonist ketamine decreases neuronal cell loss in the cortex by blocking excitotoxic damage and apoptosis during cerebral ischemia. Through the activation of the sympathetic nervous system, ketamine has the potential to maintain cerebral perfusion pressure and decrease the need for vasoactive drugs after a CPB. Additional neuroprotective effects of ketamine may be due to its ability to reduce systemic and CNS inflammation after surgery (Hudetz J. et al., 2009). Ketamine has an anti-inflammatory impact and reduces postoperative delirium (POD) after heart surgery with a CPB graft (Hudetz J. A. et al., 2009). Cardiac surgery is associated with an increased risk of postoperative cognitive dysfunction (POCD). After cerebral ischemia, ketamine protects neurons by reducing inflammation and excitotoxicity. One week after heart surgery, ketamine reduces POCD, which may be associated with the drug’s anti-inflammatory effects (Hudetz J. et al., 2009).

Researchers, in order to find out whether ketamine, when given during CABG surgery, decreases the amount of opioids needed in the aftermath of the procedure, the patient will be given a 0.5 mg/kg bolus dose of ketamine intravenously before the skin incision. They will then be given an infusion of 0.5 mg/kg/h until the procedure is completed. A total of 183 patients underwent screening, with 80 being randomly assigned. The two groups started with comparable features. Within the first 2 days after surgery, patients in the intervention group were given 53.6 mg of morphine equivalents, whereas those in the placebo group were given 55.7 mg. During the first 2 days after surgery, there were no discernible changes in the average, minimum, or maximum pain ratings nor in the morphine equivalents measured at 6, 12, or 24 h postoperatively. Postoperative pain ratings and opioid use were unaffected by ketamine administration during CABG (Cameron et al., 2020).

In pediatric open-heart surgery, researchers looked at how opioid intake during perioperative periods changed after receiving a low-dose ketamine infusion. The researcher’s study included male and female patients undergoing heart surgery who were 2–12 years old. In the ketamine group, patients were given a 0.3 mg/kg bolus before skin incision, then 0.25 mg/kg/h intraoperatively, and 0.1 mg/kg/h for 24 h postoperatively. The durations until extubation were comparable for patients in the control and ketamine groups. The ketamine group showed a substantial decrease in modified objective pain levels compared to the control group. Neither group’s subjects reported any hallucinations or diplopia. Research has shown that children having open-heart surgery benefit from a low-dose ketamine infusion, which decreases their need for opioids during and after the procedure, as well as their pain ratings after the procedure. Moreover, ketamine did not make anyone hallucinate or have diplopia (Abdelfattah et al., 2024).

The effectiveness of ketamine in reducing post-CABG delirium is still up for debate, according to recent research. Coronary artery bypass surgery is connected with a prominent complication known as POD. Postoperative cognitive impairment is common after surgical operations, especially cardiac procedures involving hypothermia and CPB pumps. Patient outcomes are greatly affected by the high prevalence of delirium after heart surgery, which reaches 90%. Due to the elderly patient demographic and unique factors associated with the use of CPB pumps, which need specialized anesthetic procedures, cardiac surgery-related delirium differs from POD observed in other surgical situations. A number of studies have cast doubt on the safety and efficacy of ketamine, while others have shown that it may be an effective tool in the fight against delirium. In order to improve postoperative recovery while reducing hazards, further study in this field is required. At this time, doctors need to be up-to-date on the newest research and thoroughly assess the pros and cons of ketamine for each patient. The next step in maximizing the advantages of ketamine while minimizing its hazards is to determine the best way to administer the drug, when to provide it, and how to classify patients. Clinicians, taking into account patient characteristics and the larger therapeutic context, must carefully weigh the possible benefits of ketamine therapy against its likely side effects until such data accumulates. Therefore, while ketamine shows potential as part of multimodal approaches to reducing POD, it should only be used after careful consideration based on the most recent scientific findings and adapted to the unique needs of each patient. To better understand and use ketamine in the perioperative setting of patients having CABG surgery, more research in this area is not only recommended but considered essential (Maroufi et al., 2024).

POD and POCD are expected consequences of thoracic surgery, especially in individuals aged 65 and older. These issues may impact recovery and healthcare expenditures to a greater or lesser extent. This research looks at how this population responds to low-dose S-ketamine in terms of POD and POCD reduction. Highlighting its neuroprotective potential, low-dose S-ketamine substantially lowers the incidence of POCD in patients aged 65 years and above after thoracic surgery. In order to enhance postoperative outcomes and decrease healthcare expenditures in this particular patient group, researchers supported the use of S-ketamine in anesthetic regimens (Wang et al., 2024).

### 2.4 Opioid agents in patients undergoing cardiac surgery

The more well-known opioids, including morphine, hydromorphone, and fentanyl, are used as perioperative anesthetic agents. During the intraoperative time, less famous but far more powerful opioid medicines like alfentanil, remifentanil, and sufentanil are intravenously administered continuously. The preferred intraoperative opioid agent for many years has been fentanyl due to its quick onset, high potency, and ease of dosage for anesthesiologists (Sridharan and Sivaramakrishnan, 2019; Jabalameli et al., 2011).

Since the inception of cardiac surgery, opioids have been necessary in the fields of anesthesia and analgesia. Opioids are still used most often for pain relief after heart surgery, although there is a lot of variation in the type, dosage, and administration method. Following a brief overview of opioid usage in cardiothoracic anesthesia, this section compares and contrasts the various opioids currently in use and crucial outcome variables in cardiac anesthesia, including postoperative analgesia, extubation times, fast-track cardiac anesthesia, chronic neuropathic pain, and cardioprotection (Kwanten et al., 2019).

Although there are many factors contributing to the opioid problem, one acknowledged risk factor is surgical procedures that expose patients to opioids for the first time. On top of that, people tend to take more opioids after surgery than is necessary, even when they do not feel any pain. Patients after cardiac surgery had rates comparable to those observed in smaller studies, and new chronic opioid usage occurs in 3%–10% of patients following minor and significant general surgical operations, according to recent research. An epidemic of chronic opioid addiction has emerged as a result of the excessive use of opioids for the treatment of acute pain. Researchers quantified the number of opioid-naive patients who acquire a tolerance for opioids after heart surgery to examine whether there is a correlation between the first dosage of opioids given to patients at discharge and the risk of establishing a new addiction. Within 90–180 days following surgery, researchers found out what percentage of individuals who had never used opioids before acquired new chronic opioid usage. Researchers computed oral morphine equivalents (OMEs) for the first opioid prescription filled upon release. The OMEs at discharge and the chance of developing chronic opioid usage were analyzed using a multivariable logistic model with cubic splines. After cardiothoracic surgery, opioids are often utilized, and approximately 10% of patients will keep using them for more than 90 days after the procedure. Persistent usage was also strongly linked with greater OMEs, which were recommended at discharge. Researchers suggested that clinics should implement measures to educate patients more and reduce opioid prescriptions once they leave the facility (Kwanten et al., 2019).

There is some evidence that long-term opioid usage increases the risk of CVD, although how exactly opioids contribute to the development of this condition remains unknown. Persistent opioid usage has been linked in a small number of studies to many cardiovascular complications, including coronary heart disease, myocardial infarction, poor perfusion after myocardial infarction, and mortality from these causes. There is strong evidence that infected endocarditis (IE), which accounts for 5%–10% of fatalities in the intravenous drug users (IVDU) group, contributes to overall mortality in this population. There has been a noticeable increase in the number of patients with opioid dependence or continuous usage who have undergone heart surgery in the last 10 years. There is no risk to the patient during cardiac surgery if they have an opioid use disorder (OUD), but there is an increased risk of complications and costs. Patients with OUD should not be denied surgery, but they should be closely monitored for any issues that may arise after the procedure (Dewan et al., 2019). Furthermore, patient- and drug-related factors increase the likelihood of continued opioid usage after surgery. These findings may help guide efforts to reduce overprescribing of opioids to patients undergoing cardiothoracic surgery. To address this, further studies are required to clarify community-based prescription procedures for these patients and identify the variables that contribute to prolonged opioid use continuing beyond the anticipated period (Kurteva et al., 2024).

Clinician researchers have several critical responsibilities in the future. So far, there have only been case reports and tiny subsets of observational cohorts demonstrating the use of OFA and analgesia in the context of cardiac surgery. This is despite the fact that there are several alternatives to opioids and the opportunities they provide. In order to obtain more support, opioid-free (or even opioid-sparing) techniques should find and promote algorithms that are just as effective as the current strategy but have better safety profiles. They also need to prove that they are feasible. It must also be acknowledged that opioids do not necessarily equate to harm. Opioids may still be a valuable tool for controlling pain during surgery if taken as prescribed, which is to alleviate unbearable pain that cannot be relieved by other methods and for the shortest feasible time at the lowest effective dosage. CPB intervention, minimally invasive procedures, transcatheter technologies, indwelling mechanical support devices, and many other innovations have contributed to the long and fruitful history of cardiac surgery. When it comes to creating and implementing perioperative pathways, the field has long been at the forefront of innovation. The available evidence of opioid harm, the presence of effective non-opioid alternatives, and interdisciplinary frameworks call for the development of research strategies and the launch of novel clinical programs to reduce opioid use and re-evaluate their role in cardiac surgery (Grant et al., 2021).

Analgesia is necessary for both the immediate alleviation of pain and the prevention of chronic discomfort after heart surgery, which peaks within 48 h after the procedure. Despite the hazards, including side effects and reliance, opioids are often prescribed for postoperative pain treatment. Opioid dependency is a concern after heart surgery since pain might originate from somatic, visceral, or neuropathic sources. The use of multimodal analgesia, which incorporates many drugs and regional anesthetic procedures, is being advocated as a means to reduce opioid usage and the complications it entails. A few examples of strategies include acetaminophen, gabapentinoids, intravenous lidocaine, anti-inflammatory medications, alpha-2 agonists, and NMDA antagonists. Better pain management, less opioid dependence, and better outcomes after heart surgery are all possible with these methods. When it comes to improving patient recovery, ERAS^®^ Cardiac (the Society for Enhanced Recovery After Cardiac Surgery (ERACS)) is a staunch champion for a multimodal strategy that minimizes opioid use in order to decrease problems and increase patient satisfaction. There is evidence that multimodal pain therapy after cardiac surgery (CS) improves outcomes compared to conventional care. This approach is both practical and reasonable. In a cardiac surgery scenario where surgical techniques and device technology are constantly evolving, the perioperative analgesia strategy needs to keep up with these changes to ensure faster patient recovery, adequate pain control without the common adverse side effects of opioid-based analgesics, and a safe and effective operation. There is less evidence for ketamine infusion, but gabapentinoids, acetaminophen, low doses of dexamethasone, dexamethasone, and nonsteroidal anti-inflammatory drugs (NSAIDs) (in selected cases), and methadone appear to be the most effective opioid-sparing drugs for post-cardiac surgery pain control. Methadone has opioid-related side effects. While spinal anesthesia, epidural, and paravertebral blocks have all observed a decline in use due to safety concerns, ultrasound-guided fascial plane blocks have proven to be a safe and effective alternative. The ERACS now recommends including these blocks into a multimodal analgesia strategy for patients undergoing cardiac surgery (Fernandes et al., 2024).

### 2.5 Inhaled anesthetic agents in patients undergoing cardiac surgery

To induce and maintain general anesthesia in the operating room, inhalation anesthetics are used. The most often used agents in practice today are nitrous oxide, halothane, isoflurane, desflurane, and sevoflurane. By lowering systemic vascular resistance, isoflurane, sevoflurane, and desflurane all lower systemic BP. Typically, these medications maintain cardiac output; nevertheless, cardiac depression may occur when used in conjunction with other intravenous drugs or in individuals with severe cardiogenic shock (Okamoto et al., 2015; Miller et al., 2020).

It is now impossible to completely rule out the possibility of occupational risks resulting from operating room staff being exposed to inhaled anesthetics. Researchers assessed whether operating room staff members’ occupational exposure to waste anesthetic gases during CPB complies with the set regulatory limitations. Nitrous oxide and either desflurane (n = 5) or sevoflurane (n = 5) were used to provide inhalational anesthesia during CABG in 10 people. Prior to starting CPB, inhalational anesthetics were no longer administered. Before and during CPB, gas samples were taken every 90 s from the anesthesiologist’s (A), surgeon’s (S), and perfusionist’s (P) breathing zones. Ample air conditioning and regular use of waste gas scavenging systems on CPB equipment are two ways to further reduce occupational exposure to inhalational anesthetic agents, although occupational exposure was generally low during the study, and none of the operating room staff reported subjective or objective impairment or discomfort (Mierdl et al., 2003).

CPB and myocardial revascularization both carry the risk of IRI, which may harm end organs like the heart. Researchers showed that volatile anesthetics preserve the myocardium. Because of the adverse effects of propofol, which limits myocardial protective actions when administered in conjunction with other drugs, it remains unclear whether this offers any practical advantage. Timely, well-accepted, and fully completed recruitment of higher-risk patients having CABG surgery to a trial comparing total inhalational and propofol anesthesia is feasible (Milne et al., 2024).

Due to a lack of intravenous anesthetics during the COVID-19 epidemic, researchers had to come up with novel ways to perform heart surgery without them. Because some vaporizers were unavailable for providing inhaled drugs during CPB in the hospital, researchers used an anesthetic-conserving device (Sedaconda-ACD) to provide complete inhaled anesthesia. The researchers recorded experience and postoperative cardiovascular outcomes. Troponin’s peak level was the primary endpoint, whereas other cardiovascular problems were the secondary outcome. Researchers did not observe any increase in postoperative problems in the sample, and the Sedaconda-ACD device allowed researchers to induce anesthesia without the need for intravenous drugs. Sevoflurane-assisted total inhaled anesthetic did not correlate with a decreased risk of myocardial damage as measured by the postoperative troponin peak level. However, there was less inotropic medication usage in the researchers’ population (Labaste et al., 2024).

Using a systematic review and meta-analysis, researchers assessed the safety and effectiveness of volatile anesthetic for postoperative sedation in adults undergoing heart surgery. Although there is no difference in ICU or hospital stay duration, volatile anesthetic sedation may be linked to a quicker time to extubation after heart surgery. The effect on unfavorable cardiovascular outcomes is unclear. However, it is related to a significantly reduced postoperative troponin concentration. There must be randomized, blinded studies that use intention-to-treat analysis (Spence et al., 2017).

Because of their cardioprotective properties, volatile (inhaled) anesthetics may help patients undergoing CABG achieve better clinical results. After 1 year, anesthesia with a volatile agent did not significantly reduce the number of fatalities among patients undergoing elective CABG compared to complete intravenous anesthesia (Landoni et al., 2019). Furthermore, when it came to the incidence of short-term POCD, inhalation anesthetic was just as common as intravenous anesthesia for older patients having non-cardiac surgeries. The neurological system may be more severely damaged by inhalation anesthetic, and there is no difference in the amount of time it takes for cognitive function to return after 5–7 days (Huang and Zhang, 2023).

### 2.6 Muscle-relaxant drugs in patients undergoing cardiac surgery

There are several uses for muscle relaxants in anesthesiology. They may be used to enhance mechanical breathing, safely intubate patients, or get patients ready for surgery. The mode of action of muscle relaxants may be divided into two categories: centrally acting muscle relaxants and depolarizing and non-depolarizing muscle relaxants. Nicotine receptors are competitively antagonistic to non-depolarizing neuromuscular blocking drugs (NMBDs), such as tubocurarine, atracurium, pipecuronium, mivacurium, pancuronium, rocuronium, and vecuronium. By doing this, these medications prevent acetylcholine’s depolarizing impact, which eliminates the possibility of muscle fiber activation. Depolarizing medications such as succinylcholine and decamethonium first activate (depolarize) the receptor, followed by a gradual and long-lasting blockage. Unlike acetylcholine, these medications work as more persistent agonists rather than competing antagonists. The length of time these medications work might be affected by a variety of circumstances. Among them, changes in the acid–base balance and electrolyte imbalance may be significant. Alkalosis causes resistance to the actions of non-depolarizing muscle relaxants, while acidosis improves their efficacy. Alkalosis and acidosis have opposing effects in depolarizing medications. Research on how disruptions in acid–base balance affect non-depolarizing relaxants has produced contradictory findings (Radkowski et al., 2024).

The non-depolarizing neuromuscular blocking drugs such as pancuronium, vecuronium, rocuronium, and mivacurium all have varying degrees of effect on the cardiovascular system. Their impact on the cardiovascular system is essential to consider while administering anesthesia to patients, especially those having heart surgery. Scientists held mice in organ pools after removing their left or right atriums. Researchers monitored changes in heart rate (HR) in correct atrial fibrillation (AF) while administering desipramine (10^–7^ M), the nonselective β-blocker propranolol (10^–8^ M), or pancuronium, vecuronium, rocuronium, or mivacurium (10^−9^–10^–5^ M) in both the presence and absence of these drugs. A bipolar platinum electrode was used to stimulate left atrial preparations with electrical field stimulation. The effects of different concentrations of non-depolarizing neuromuscular blocking agents (NMBAs) on the developed force were recorded, both with and without propranolol (10^–8^ M) and desipramine (10^–7^ M). When compared to the control group, pancuronium caused a dose-dependent increase in HR. A dose-dependent increase in HR was also observed with vecuronium, rocuronium, and mivacurium, but the effects were not statistically significant, according to the researchers. The impact of pancuronium on HR was reduced by propranolol, whereas the effects of vecuronium, rocuronium, and mivacurium on HR were unaffected. Neither vecuronium, rocuronium, mivacurium, nor pancuronium was affected by desipramine in terms of HR. At doses of 10^–5^ M for pancuronium and 10^–6^ and 10^–5^ M for vecuronium, rocuronium, and mivacurium, respectively, researchers demonstrated that all four medications enhanced generated force in a dose-dependent manner. The inclusion of propranolol eliminated these increases in developed force. Desipramine had no impact on the cumulative force effects of the four medications. The direct activation of β-receptors may cause the HR effect of pancuronium and the developed force effects of vecuronium, rocuronium, mivacurium, and pancuronium. Despite research being *in vitro*, the results might have significant implications for pathologic conditions like hypertension, where β-blocking medicines are often used by patients and may lead to an increase in β-receptor expression (Gursoy et al., 2011).

In another trial, 66 patients having elective valve surgery were randomly assigned to receive 0.6 mg/kg of rocuronium bromide (Group R, n = 22), 0.1 mg/kg of pancuronium bromide (Group P, n = 22), and 0.1 mg/kg of vecuronium bromide (Group V, n = 22). Researchers recorded HR and arterial pressure measurements (systolic, diastolic, and mean) at the following phases: (1) baseline, when hemodynamics remained steady for 2 minutes after anesthesia induction; (2) 1, (3) 3, and (4) 5 minutes following muscle relaxant administration; and (5) 1, (6) 3, and (7) 5 minutes following intubation. Five minutes after receiving a muscle relaxant injection, group R’s HR decreased from 93.9 ± 21.3 beats per minute to 82.4 ± 20.7 beats per minute. After intubation, it increased to 128.3 ± 25.8 beats per minute, but 5 minutes later, it returned to baseline. One minute after receiving a pancuronium injection, group P’s HR increased from 98.8 ± 32.6 to 109.6 ± 32.7 beats/min, and this increase continued for the duration of the trial. Five minutes after the medication injection, group V’s HR decreased from 99.9 ± 22.3 to 83.8 ± 19.6 beats per minute. One minute after intubation, it increased to 118.6 ± 22.4 beats per minute, and 5 minutes later, it returned to baseline. Systolic, diastolic, and MAP decreases were significantly correlated with the HR decrease in groups R and V. Only the systolic BP in group P showed a substantial decrease 5 minutes after medication administration. All groups observed a significant increase in systolic, diastolic, and MAP upon intubation. All three medications showed excellent intubation circumstances (intubation score 3–4). Nonetheless, some patients in group P displayed diaphragmatic movement while being intubated. Rocuronium has the quickest start of muscle relaxant activity. In conclusion, individuals with regurgitant lesions who have slower baseline HRs should utilize pancuronium since it significantly increases HR. Vecuronium and rocuronium, according to researchers, have lower HRs and should be selected for individuals whose baseline HRs are higher. Both rocuronium and vecuronium provide optimal intubating conditions; however, rocuronium has a faster onset (Virmani et al., 2006).

Patients in need of invasive mechanical ventilation due to acute myocardial infarction (AMI) are among the sickest and most dangerous patients in the medical system. NMBAs are often needed for quick sequence induction during endotracheal intubation. Some studies have shown that succinylcholine improves first-attempt success, but others have shown comparable results with rocuronium. Thus, it is still unclear whether NMBA is better. Previous research on NMBAs for induction has not included many people who had primary CVD. Ultimately, when comparing succinylcholine to rocuronium for patients with AMI who need IMV, the latter may be linked to lower in-hospital death rates. The investigators believed that a randomized controlled trial with the correct dosage of rocuronium was required to evaluate outcomes in disease-specific populations, like those with AMI, before making any changes to practice; this is due to the unique physiological features of these patients and the fact that our results should only be viewed as hypothesis-generating (Schenck et al., 2023).

### 2.7 Adjunct medications in patients undergoing cardiac surgery

In order to reduce sympathetic reflex tachycardia and hypertension, the cardioselective β-adrenergic antagonist esmolol is administered during general anesthesia. Researchers demonstrated that during the intubation phase, the systolic BP in the younger patients increased to 100%. In contrast, in the older patients, it stayed stable at approximately 89% of the pre-induction levels. While younger patients’ diastolic BP increased to 107% of their pre-induction levels during the same period of anesthesia, older patients’ stayed at approximately 91%. Both groups of patients used the same amount of medication and recovered from anesthesia at the same rate and quality. Esmolol infusions support the idea of general balanced anesthesia for both younger and older patients undergoing elective upper abdominal surgeries (Lončar-Stojiljković, 2021). Since cardioplegic solutions save the heart from ischemia during aortic cross-clamping and stop its electro-mechanical activity, they constitute the gold standard for myocardial protection during cardiac surgery. However, myocardial injury has a significant clinical consequence. Researchers investigated the theory that by essentially lowering myocardial activity and, thus, oxygen consumption to 0, the short-acting beta-blocker esmolol, given both immediately before and as a cardioplegia supplement, would provide additional protection to myocardial tissue during CPB. Esmolol lowers peak postoperative troponin levels in high-risk individuals undergoing elective heart surgery with cardioplegia. To determine the impact of esmolol on significant clinical outcomes, further research is required (Bignami et al., 2017).

Patients may experience a decrease in quality of life, extended stays in critical care and hospitals, and higher medical expenses as a result of the most prevalent problems that arise after heart valve surgery, which include perioperative myocardial injury/infarction, arrhythmias, and heart failure. The prognosis of patients having heart valve surgery may be improved by reducing the perioperative problems. Analgesic, sedative, anti-sympathetic, and opioid-sparing are some of the effects of DEX, a potent and very selective alpha-2 adrenoceptor agonist. DEX is an effective supplementary agent to cardiac anesthesia, according to many investigations. Myocardial ischemia and other postoperative complications in cardiac surgery can be lessened with the use of DEX. This anesthetic adjuvant decreases the need for opioids, inhalation anesthetics, and intravenous anesthetics. Its anti-sympathetic action lowers myocardial oxygen consumption by reducing metabolism and preventing tachycardia (Lee, 2019; Taittonen et al., 1997; Fan et al., 2023). Researchers focused on cardiac surgery using DEX as an anesthetic adjuvant, aiming to determine its association with perioperative problems and cardiothoracic ICU LOS. Researchers indicated that significant postoperative complications are related to patient age, length of CPB, and duration of mechanical ventilation in patients having heart valve surgery. Prolonged stays in the critical care unit are linked to factors such as advanced age, preoperative NYHA classification 4, diabetes mellitus, intraoperative bleeding, and red blood cell transfusion. DEX, given intraoperatively, has the potential to enhance these types of clinical results (Fan et al., 2023).

To modulate the hemodynamic response to tracheal intubation in patients with coronary artery disease, researchers examined the effects of DEX, esmolol, and a combination of the two. Patients undergoing elective CABG benefited from the combination of DEX and esmolol on HR and pulmonary arterial pressures. Still, there was no additional benefit on arterial BP compared to the DEX and esmolol groups (Singh et al., 2019).

Nitroglycerin has been around for almost a century and is still widely used today. Historically, it has been administered under the tongue to alleviate the symptoms of angina and congestive heart failure by widening the coronary arteries. Some of the newer vasodilator medications include phosphodiesterase inhibitors, autonomic ganglion blockers (such as pentolinium and trimetaphan), calcium channel blockers (such as diltiazem and verapamil), ARBs, ACE-inhibitors, direct-acting arterial vasodilators (like hydralazine and nitroprusside), and minoxidil and papaverine, among others. Both acute and chronic cases of hypertension may benefit from these medications. The majority of these drugs mainly target arterioles by triggering the smooth muscle of blood vessels to widen. The endothelium of blood vessels has been the subject of increased study throughout the last 20 years. Every cell in the human body receives oxygen and nutrients from this enormous organ (Thangathurai and Hong, 2023).

When planning any surgery, the anesthesiologist must keep cardiovascular protection in mind. In most cases, this is the case when the patient poses a significant risk of coronary or vascular involvement during surgery; nevertheless, this does not always complicate the surgical approach. After all, patients already face perioperative risks associated with anesthesia. When treating coronary disease, nitroglycerin is a common vasodilator. One of the leading causes of complications and deaths during surgery is myocardial ischemia. The use of the vasoactive drug nitroglycerin in patients under anesthesia has had very mixed outcomes. Patients with severe hemodynamic or cardiovascular impairment, however, have benefited from it. If administered correctly and not for an extended period, nitroglycerin may have a cardioprotective impact on the anesthetized patient. Furthermore, it is essential to take into account the potential adverse effects on the patient. Every instance should be assessed individually, with the benefit being interposed but not used routinely, particularly in patients with severe hemodynamic impairment (Thangathurai and Hong, 2023).

Myocardial ischemia ranks high among the many potential problems that could arise after cardiac surgery. When it comes to heart surgery, calcium channel blockers and nitroglycerin are often employed since they provide some protection against myocardial ischemia. A meta-analysis compared nitroglycerin and calcium channel blockers for their effects on myocardial protection, HR, and BP. In terms of protecting the heart, the meta-analysis finds no statistically significant difference between nitroglycerin and calcium channel blockers; nevertheless, the nitroglycerin group had a faster HR (Lai et al., 2024).

When it comes to the pharmacologic therapy of the low-output syndrome after CPB, milrinone and dobutamine are both suitable and equivalent (Feneck et al., 2001). Researchers evaluated milrinone in comparison to dobutamine and nitroglycerin for the treatment of severe pulmonary hypertension in young patients having mitral valve replacement. Forty individuals were set up for elective mitral valve replacement because their systolic pulmonary arterial pressure was 60 mmHg or higher. Based on the medications administered, the patients were randomly split into two equal groups. A loading dosage of 50 μg/kg over 10 min and a maintenance dose of 0.25–0.75 μg/kg/min of milrinone were administered by researchers to patients in group II, whereas patients in group I received dobutamine at a rate of 5–20 μg/kg/min and nitroglycerin at a rate of 0.5–3 μg/kg/min. Researchers indicated that by reducing pulmonary artery and pulmonary capillary wedge pressures to a more significant extent, milrinone ensures sufficient cardiac function (Eskandr et al., 2018).

**FIGURE 1:**
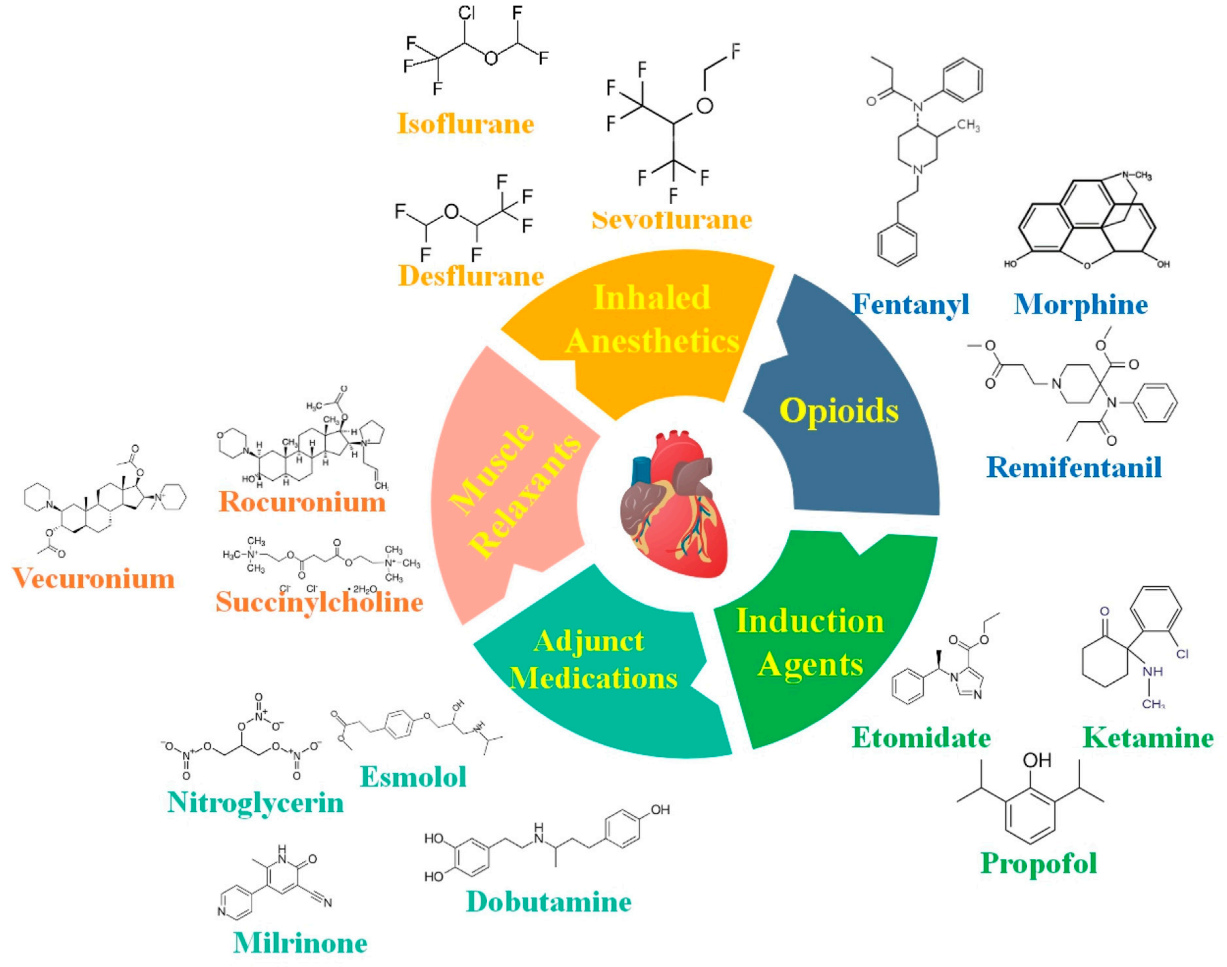
Anesthetic medications are carefully selected for cardiovascular operations to ensure steady circulatory function while providing adequate anesthesia. Some of the most critical anesthetic medicines and factors to consider while caring for patients with CVDs are provided in this figure.

When a patient presents with cardiogenic shock, inotropes are an essential part of stabilizing them early on. The two most popular inotropes, milrinone and dobutamine, have been used in clinical settings for many years, but there is limited evidence comparing their effects. To determine whether milrinone or dobutamine was safer and more successful for the first treatment of cardiogenic shock, researchers performed a retrospective study. The researchers’ study included 50 adult patients who had first inotrope treatment with milrinone or dobutamine for cardiogenic shock and 50 patients who did not receive mechanical circulatory support, regardless of the origin of the shock. The duration until cardiogenic shock resolved was the primary end goal. Patients treated with milrinone were just as likely to experience hypotension as those treated with dobutamine (49.2% vs. 40.3%), but arrhythmias were more prevalent in the dobutamine group. There was no difference between the groups with regard to the use of concurrent vasoactive drugs, the necessary dose, or the length of treatment. The total incidence of cessation owing to adverse events was not different; however, dobutamine was usually stopped more often for arrhythmia and milrinone for hypotension. Although their adverse event profiles differed, milrinone and dobutamine were both shown to be efficacious and safe. Researchers suggested that the tolerance of adverse effects should be the primary consideration when choosing between milrinone and dobutamine as the first inotrope treatment for cardiogenic shock (Lewis et al., 2019) (Table 1; Figure 1).

**TABLE 1:** Anesthetic drugs commonly used and their considerations in cardiovascular settings.

Anesthetic drug	Class of drugs	Effect on the cardiovascular system	Reference
Etomidate (ETO)	Induction agent	Research has shown that in healthy individuals, low dosages of ETO administered during anesthesia induction result in few changes to the heart rate (HR) (<10%). However, other hemodynamic variables such as cardiac index, systemic vascular resistance, central venous pressure, and pulmonary artery pressure remain unchanged	Valk and Struys (2021b)
Propofol	Induction agent	One type of intravenous anesthetic is propofol, which is chemically distinct from all the others. Unlike other anesthetic drugs, it may be safely used for the induction and maintenance of anesthesia throughout most surgical operations. It is beneficial for sedation in the postoperative environment or critical care unit. According to clinical trials, the use of propofol in conjunction with opioids is a rational option for anesthesia in heart surgery	Searle and Sahab (1993)
Ketamine	Induction agent	Because of the potential for dose-dependent elevations in blood pressure (BP), HR, and cardiac output, ketamine should be administered with care to patients with cardiovascular problems, including hypertension or heart disease. Additionally, it can cause hypertension, arrhythmias, and tachycardia	Goddard et al. (2021)
Fentanyl	Opioid	As an analgesic (pain reliever) and anesthetic, the FDA has authorized the use of fentanyl, a powerful synthetic opioid medication. When compared to heroin and morphine, its analgesic effects are almost 100 times stronger	Armenian et al. (2018)
Remifentanil	Opioid	A potent, short-acting synthetic opioid analgesic, remifentanil is sold under the brand name Ultiva. In addition to the anesthetic agent, it is administered to patients to alleviate discomfort during surgery. Both alone and in combination with other drugs, remifentanil provides drowsiness and general anesthesia. Reduced arterial pressure, cardiac output, and systemic vascular resistance define the well-studied hemodynamic response of remifentanil	Zaballos et al. (2009)
Morphine	Opioid	Opioid pain reliever morphine is given to patients with really severe pain when other painkillers have failed or are not an option. Research has also shown that morphine lowers the cardiac output and pulse rate by depressing the myocardium	Sethna et al. (1982)
Sevoflurane	Inhaled anesthetic	During procedures and surgeries, the volatile anesthetic sevoflurane may induce hypnosis, forgetfulness, analgesia, akinesia, and autonomic blockade. Since sevoflurane does not alter filling pressures or HR and reduces the cardiac index or systemic vascular resistance, it is believed that myocardial contractility is reduced	Ebert et al. (1995)
Isoflurane	Inhaled anesthetic	One type of anesthetic is isoflurane, which goes by many names on the market. Isoflurane may irritate the airway, so other drugs are usually used to induce anesthesia instead. However, it can be used to maintain anesthesia. You inhale isoflurane to get the drug. Isoflurane maintains the cardiac output while decreasing systemic vascular resistance in a dose-dependent manner, which, in turn, lowers systemic arterial pressure and increases the HR.	Marano et al. (1996)
Desflurane	Inhaled anesthetic	Isoflurane and desflurane are inhalation agents with similar chemical structures. With a blood–gas partition coefficient lower than nitrous oxide, it allows for fast absorption and washout of the gas, which is unique for its physicochemical features. Compared to isoflurane, desflurane has a comparable effect on reducing left ventricular systolic and diastolic function. Both mean arterial BP and systemic vascular resistance are lowered in a dosage-dependent manner	Weiskopf (1995)
Rocuronium	Muscle relaxant	Both conventional tracheal intubation and fast-sequence intubation require rocuronium injection in conjunction with general anesthetic medications. This medication is also prescribed to ease muscular tension before, during, or after surgery or mechanical breathing. Rocuronium has not been shown to have any major impact on the heart in clinical trials, with the exception of minimal hemodynamic alterations	Wierda et al. (1997)
Vecuronium	Muscle relaxant	Vecuronium, a non-depolarizing drug, paralyzes skeletal muscles by attaching to the nicotinic cholinergic receptor on the motor endplate’s postjunctional membrane and competing with acetylcholine for cholinergic receptor sites. One of its claimed benefits is that it does not have any significant adverse effects on the cardiovascular system	Morris et al. (1983)
Succinylcholine	Muscle relaxant	A skeletal muscle relaxant, succinylcholine, is pronounced SUK-seh-nil-KOH-leen. It is used to ease muscular tension before, during, or after surgery or while using a breathing machine. The use of succinylcholine, a muscle relaxant, to facilitate the process of endotracheal intubation is widespread around the globe	Hager et al. (2018)
Esmolol	Adjunct medication	The beta1 receptor blocker esmolol (brand name Brevibloc) has a short half-life, an abrupt start, and, at therapeutic doses, no discernible intrinsic sympathomimetic or membrane stabilizing function	Zangrillo et al. (2021)
Nitroglycerin	Adjunct medication	Angina, or chest discomfort, maybe a symptom of coronary artery disease; nitroglycerin can help prevent this. It may also alleviate angina attacks that have already begun. Nitroglycerin is part of the class of medications known as nitrates	Sharma et al. (2024)
Milrinone	Adjunct medications	Milrinone is prescribed to provide cardiac support to individuals suffering from acute heart failure, pulmonary hypertension, or chronic heart failure. It causes vasodilation and enhances inotropy and lusitropy, as well as the heart’s contractility and relaxation	Mathew et al. (2021)
Dobutamine	Adjunct medications	One example of an inotrope is dobutamine. Strengthening the cardiac muscle is one of its benefits. Congestive heart failure is one of its indications for use. Additional uses for this medication are possible	Liu et al. (2021)

## 3 Recent advancements in anesthesia for cardiovascular surgery

Significant technological and methodological developments in the last few decades have driven a dramatic shift in the anesthesia industry, with cardiac anesthesia at the forefront of this shift. An important step forward in patient care is the automation of anesthetic administration, which has led to more efficient and scalable systems with separate closed loops for hypnosis, analgesia, and fluid management. Cardiac output monitoring is now possible because of technological advancements that analyze the peripheral artery pressure waveform. In addition, new techniques that use a BP cuff to evaluate cardiac output without invasive procedures are a vast improvement. With the advent of artificial intelligence (AI), new methods of patient care, real-time monitoring, and data-driven decision-making have entered the anesthesiology industry. The use of AI algorithms has the potential to improve accuracy and productivity, leading to better anesthetic delivery and better patient outcomes. Modern cardiac anesthesia is ever-changing due to the development of new equipment and medications and perioperative imaging techniques like three-dimensional (3D) TEE. By incorporating these state-of-the-art innovations, patient outcomes have been significantly improved, with a noticeable decrease in mortality and morbidity. A dedication to using innovation to enhance patient care and overall clinical results is defining the future of cardiac anesthesia in the face of rapidly developing technologies (Ramachandran et al., 2023; Singam, 2023; Seger and Cannesson, 2020; Durai Samy and Taksande, 2024).

ERAS protocols are multimodal perioperative treatment routes that aim to facilitate early recovery after surgical operations by decreasing the significant stress response after surgery and preserving preoperative organ performance. Preoperative consultation, nutritional optimization, consistent analgesic and anesthetic regimes, and early mobilization are the essential components of ERAS protocols. There is a large amount of data showing that ERAS procedures enhance results; nevertheless, their adoption has been sluggish since they question standard surgical theory (Melnyk et al., 2011). The ERAS heart program is the first of its kind in the US; it is a multidisciplinary, evidence-based approach to improving post-operative recovery after heart surgery. Specialty-specific improved recovery guidelines may aid standardized clinical pathways for the delivery of high-quality, evidence-based perioperative care in several branches of surgery. Although feasibility studies have been conducted on cardiac surgery, the field has been hesitant to incorporate ERAS concepts due to several specific obstacles. However, researchers thought that an ERAS cardiac program would cut down on opioid usage, GI problems, and LOS while also making patients and doctors satisfied. After a year of implementing a specific ERAS cardiac program, researchers reported the results of testing these theories. Significant improvements in perioperative outcomes were linked to initial clinical and survey data collected after the first year of a system-wide ERAS cardiac program. Researchers are sure that this value-based strategy for heart surgery may reliably shorten recovery times, lower overall costs, and boost satisfaction among both patients and medical professionals (Williams et al., 2019).

The specific difficulties of cardiac surgery include sternotomy, CPB, related coagulopathy, blood transfusion, and the need for critical care after surgery. However, ERACS may still be helpful for some individuals undergoing heart surgery. The emphasis of the ERACS Society’s earlier publications on perioperative care for cardiac surgery is on postoperative and preoperative treatment rather than the intraoperative care that anesthesiologists provide. Researchers addressed this need by investigating the role of anesthesiology in attaining ERACS, introducing the ERACS protocol to their institution in February 2020 in conjunction with heart surgery. In February 2020, the authors’ institution implemented the ERACS protocol, a multimodal opioid-sparing analgesia that included regional blocks, a hemostasis management protocol, the reversal of neuromuscular blockade (NMB), and the administration of antiemetics. This protocol was developed in collaboration with cardiac surgery and the cardiac anesthesiology division. They compared a comparable historical group of patients who had heart surgery before an ERACS procedure was established with patients who had received ERACS measurements in a retrospective file review analysis. Patients’ time to extubation, opioid intake postoperatively, ICU LOS, and the incidence of postoperative complications (e.g., postoperative nausea vomiting [PONV]), bleeding, ICU readmission, and delirium) were the primary outcomes of the research. After a string of meticulous steps is taken, the ERACS may be achieved. It should be used throughout the whole perioperative phase, from the preoperative clinic to postoperative rehabilitation, and it does not simply mean opioid-sparing analgesia and fast-track extubation. The administration of intraoperative ERACS procedures is a critical function of cardiac anesthesiologists. Crucial stakeholders include both patients and providers. To strengthen the conclusion, an enormous randomized prospective experiment is necessary (Mondal et al., 2023).

Delays in recovery and opioid-related side effects are linked to opioid-based analgesia. Opioid dependence may be lessened with the use of several multimodal pharmaceuticals. Scheduled dosing of NSAIDs, gabapentinoids, and acetaminophen is one example. It has also been shown that intravenous analgesics such as magnesium, lidocaine, ketamine, and DEX enhance postoperative pain management and contribute to the maintenance of anesthesia. When other pain relief options have been exhausted or when the need to decrease the overall usage of short-acting synthetic opioids is immediate, long-acting opioids are an essential part of pain treatment. Several studies have shown that opioid administration is reduced, and postoperative recovery rates are enhanced when administered in a bundled form. The possible short- and long-term negative consequences of both insufficient analgesia and excessive opioid treatment after heart surgery have come to the forefront of medical attention. This complete strategy for pain management in cardiac surgery using multimodal, opioid-sparing drugs is the goal of this readymade order set (Gregory et al., 2024).

Nearly all parenteral sedations and general anesthetics include the administration of intravenous fluids. In the past, sedative drugs were administered via several techniques, such as barbotage, intramuscular injection, or volatile agent inhalation. In anesthetic practice, intravenous fluid therapy aims to maintain sufficient oxygen supply and tissue perfusion while, typically, providing a fluid vehicle for medication administration. Postoperative results may be impacted by choices made about the kind and quantity of intraoperative fluids (Saraghi, 2015). Remarkably, intravenous fluid dosage during shock resuscitation is still primarily empirical. While excessive fluid prescriptions also seem to hinder oxygen delivery and harm patient outcomes, insufficient fluid intake may cause tissue hypoperfusion and exacerbate organ failure. According to recent findings, early aggressive resuscitation of critically sick patients may prevent or reverse tissue hypoxia, prevent organ failure, and enhance outcomes (Marik et al., 2011). One of the most critical aspects of postoperative rehabilitation is goal-directed fluid therapy (GDFT), which helps patients recover faster and has a positive impact on the outcome of significant surgeries (Gao et al., 2024). In a study, a majority of 106 participants (66%) were employed by teaching hospitals and came from 18 different European nations. Postoperatively, patients undergoing heart surgery were admitted to an ICU at 73% of the institutions. Perfusionists prepared the priming solution for cardiopulmonary bypass, while anesthesiologists handled the intraoperative and postoperative fluid management. While 51.5% of centers employed balanced crystalloids for cardiopulmonary bypass priming, 36.0% used a mixture of crystalloid with synthetic colloid or albumin. During the operation, 74% of centers used balanced crystalloids, 15% utilized a mix of crystalloids and synthetic colloids, and 11% utilized alternative combinations. Nearly one-third of those surveyed did not utilize colloids. Gelatin was chosen over hydroxyethyl starches and albumin when colloids were used (60% vs. 24% vs. 16%, respectively). Respondents who were also engaged in ICU therapy (73% of the total) did not alter their hydration strategy from intraoperative to ICU. Those who modified their approach included 32% who increased their use of synthetic colloids and 29% who lowered their use of natural colloids. Fluid management during heart surgery may have evolved in recent years at European facilities. These days, balanced crystalloids, synthetic colloids (mostly gelatins), and albumin are the go-to options (Protsyk et al., 2017).

Data are lacking on fluid administration guidelines for patients hospitalized in the ICU after heart surgery. Researchers compared the duration of stay in the ICU after heart surgery using a standard procedure known to decrease fluid administration versus standard care. The quantity of fluid supplied was lowered according to a protocol-guided method that used stroke volume fluctuation to direct the administration of bolus fluid. However, there was no reduction in the duration of stay in the ICU compared to standard treatment, either until discharge or up to 24 h (Parke et al., 2021).

Because they do not cause hemodynamic alterations like neuraxial blockades, chest wall blocks such as pectoralis fascial (PECS), Systolic arterial pressure (SAP), erector spinae plane (ESP), and paravertebral (PVB) blocks are gaining popularity. The ineffectiveness of the chest wall blocks in reducing discomfort in the internal mammary area is a significant drawback of these devices. In response to these limitations, sternal blocks like the parasternal intercostal nerve block (PSINB) and the thoracic transverse muscle plane block (TTMPB) were developed to numb the anterior branches of the T2–T7 intercostal nerves consistently. In the context of anticoagulation, deep peripheral blocks are likely to be harmful. In contrast, superficial blocks are likely to be safe, according to the recommendations of the American Society of Regional Anesthesia (ASRA). Some specialists have argued that the ESP block is superficial, although it has not been formally classified as either deep or superficial. At this time, researchers are unaware of any extensive randomized controlled trials that have compared several fascial plane blocks to determine which one is best. The use of regional methods as part of a multimodal pain treatment regimen produces considerable analgesia. The aforementioned regional approaches may have more of a track record in fields other than cardiac surgery. Still, the evidence we have so far indicates they might be pretty helpful in a variety of cardiac procedures. Further studies with more extensive samples are needed to confirm the validity of the existing results, determine the safety profiles, and outline the processes of the newer regional approaches since the sample sizes of the studies that were evaluated were primarily microscopic (Jiang et al., 2021).

There has been a resurgence of interest in regional anesthesia due to its opioid-sparing benefits, which aligns with the growth of expedited recovery options after cardiac surgery. Almost every surgical specialty is working toward this paradigm shift, aimed at optimizing resource allocation and accelerating postoperative extubation and mobilization. Concerns about the safety of anticoagulated patients have limited the use of traditional neuraxial procedures in cardiac surgery. Ultrasound-guided thoracic wall blocks, however, present a promising alternative to epidurals and landmark-driven blocks between the vertebrae and intercostal spaces. Non-opioid pain management strategies have emerged recently, and although expertise in this field is currently limited, available research suggests their use will expand and may become crucial for improving recovery pathways after heart surgery (Balan et al., 2021).

### 3.1 Transesophageal echocardiography and real-time imaging in cardiovascular anesthesia

Heart valve and structural treatments have persisted as surgical operations for many decades. Imaging these structures before and after surgery became a supplementary technique to augment surgical visualization as it allows direct observation of the area of interest throughout the procedure. There have been tremendous advancements in percutaneous structural heart interventions (SHIs) that use catheters in the last 20 years. Imaging is essential to the effectiveness of these treatments because of their “blind” aspect. The primary purpose of fluoroscopy in percutaneous cardiac SHIs is to aid with the visual inspection of devices and catheters. However, seeing structures inside the heart’s soft tissues is essential for these operations to be successful. TEE has become a vital tool for guiding all percutaneous SHI procedures because of its portability and quickness in showing heart anatomy online. Although transcatheter aortic valve replacement relied on TEE in the beginning, pre-procedural cardiac computed tomography (CT) has now mostly supplanted it as the gold standard for assessing the valve size. Due to recent advancements in echocardiography, TEE can now provide real-time, 3D imaging of heart structures, simulating surgical anatomy. In addition to displaying actual 3D structures inside the heart, 3D-TEE may also show how devices and catheters within the heart interact with structures in the soft tissue around the heart, thereby acting as an additional set of eyes for the operator. In these types of treatments, real-time 3D-TEE is now a crucial adjunct to biplane and multiplane 2D TEE. The use of 3D-TEE imaging during SHI offers several advantages, such as improved device landing zone localization, better navigation throughout the heart chambers, and more straightforward identification of anatomical areas of interest. The need for suitable and consistent intraprocedural guiding suggestions is paramount due to the anticipated dramatic increase in the utilization of this technology during transcatheter treatments. The practical benefits of 3D-TEE in this setting, such as shorter procedure times, tremendous procedural success, fewer procedural problems, and lower radiation and contrast exposure, can only be shown by future large-scale prospective investigations (Farina et al., 2023).

As a result of a collaborative effort between the two organizations, standards for perioperative TEE training, practice, and quality improvement have been developed. A growing number of cardiac point-of-care ultrasonography (POCUS) procedures are becoming standard practice for anesthesiologists. There are presently no established standards for the training, certification, or practice of perioperative transthoracic echocardiography (TTE) by anesthesiologists, although the National Board of Echocardiography established a unique certification in “Critical Care Echocardiography” in 2019. The authors provide a suggested order for conducting an examination adapted to the assessment of perioperative patients and a description of the types, indications, and uses of perioperative TTE. No PTTE standards have been reported in the literature. Researchers suggested that in order to set standards, the American Society of Echocardiography and the European Society of Cardiology must collaborate with the Society of Cardiovascular Anesthesiologists and the European Association of Cardiothoracic Anesthesiologists, both of which are cardiac anesthesia societies (Subramaniam et al., 2022).

The most recent recommendations call for all comprehensive exams to use 3D TEE. According to the researchers’ study, almost all mitral valve operations and transcatheter procedures used intraoperative 3D TEE, but only 50% of the other valve surgeries and treatments used this technology. Reasons for its utilization might include the scarcity of time for TEE procedures, the availability of 3D equipment, and qualified staff. Educational programs that teach its use may accelerate the widespread adoption of 3D TEE (Schemberg et al., 2024). In addition to performing hemodynamic and functional monitoring of the cardiovascular system, it allows anesthesiologists to see the anatomical architecture of the heart and major arteries (Akiyama et al., 2013).

**FIGURE 2:**
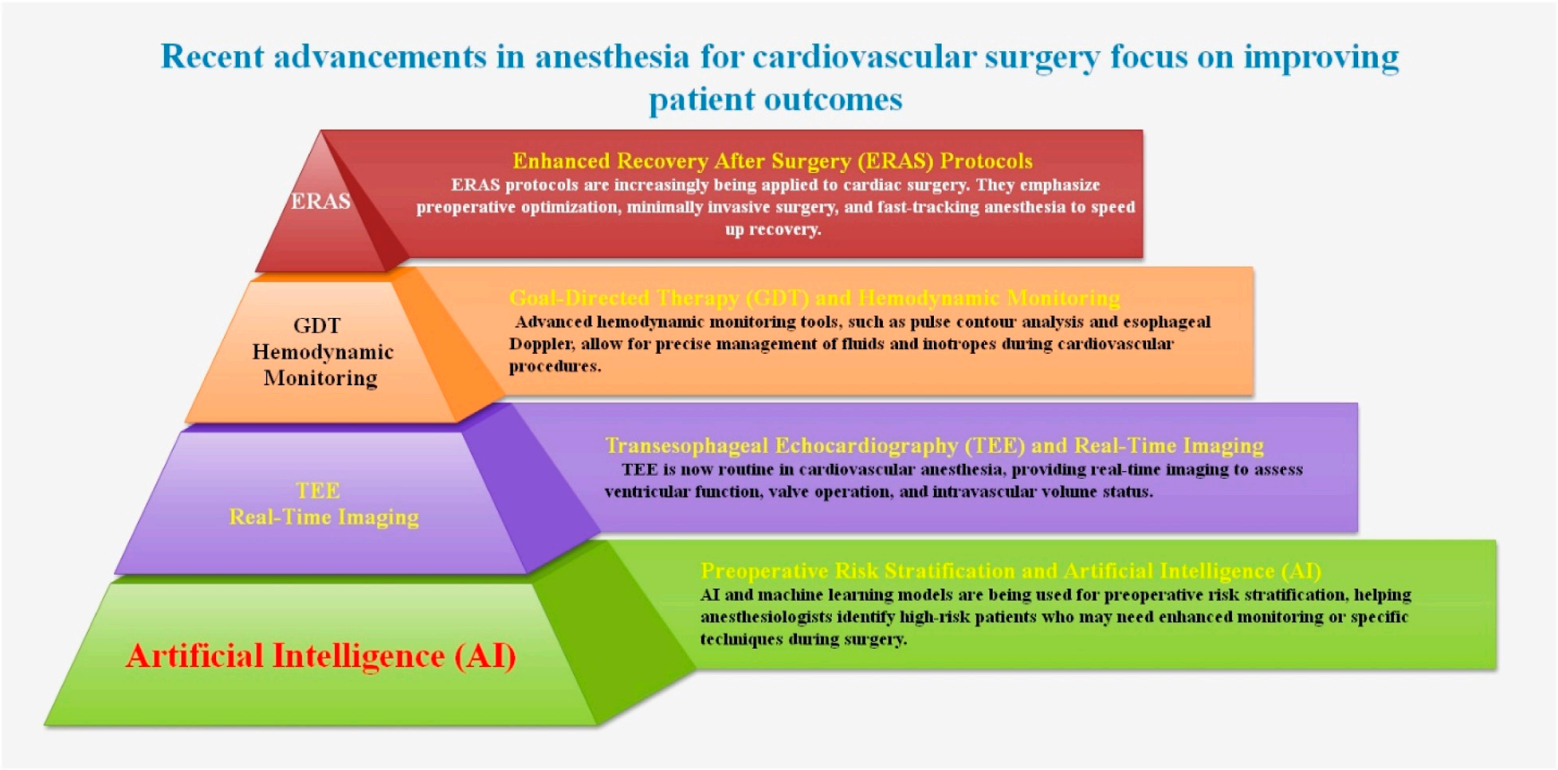
This illustration shows some recent techniques and approaches in cardiovascular anesthesia. Recent advancements in anesthesia for cardiovascular surgery focus on improving patient outcomes through precision and reducing risks associated with cardiac procedures.

In terms of world health, CVDs pose a significant problem. Heart disease and stroke have been on the rise recently, especially in the US, despite significant advancements in prevention, diagnosis, and treatment. Perioperative TEE has emerged as a critical tool in cardiac surgery because of the ever-increasing success rates of the procedure and the increased safety it provides to patients. Before, during, and after complex surgeries like mitral valve repair or aortic valve replacement, TEE is an essential tool for planning, monitoring, and evaluating the outcome of the operation. On the other hand, AI opens up new possibilities for TEE picture processing and diagnostic assistance, which may significantly enhance diagnostic accuracy and decision-making skills in real time. High expenses, unequal technical dissemination, and the high skill requirements for medical staff are some of the problems that arise while using TEE technology. Thus, it is essential to develop uniform training regimens and enhance cooperation throughout disciplines (Liang et al., 2024) (Figure 2).

## 4 Recent developments in improving anesthesia management, specifically in patients with cardiovascular disease

**FIGURE 3:**
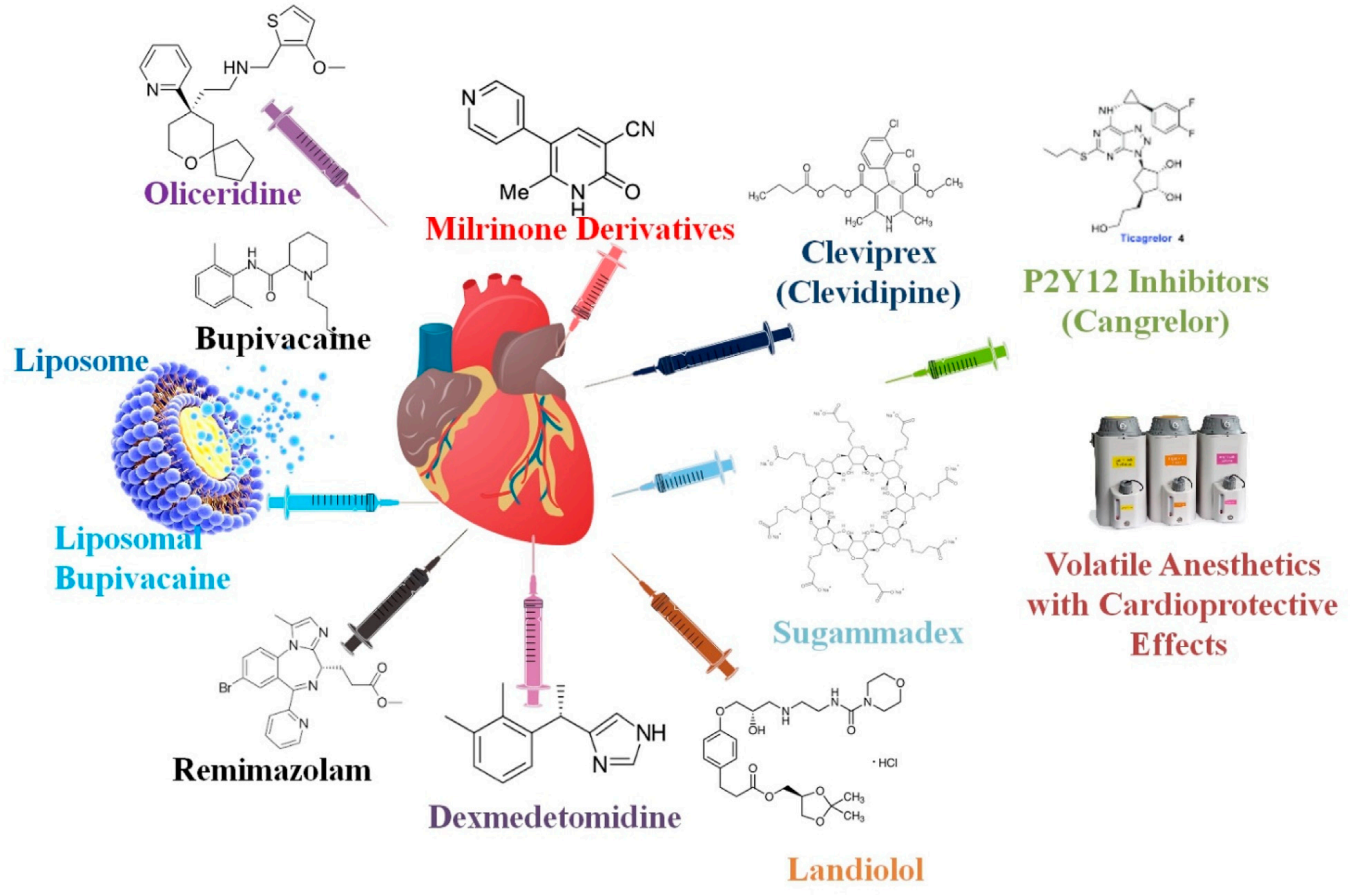
Novel medications and methods have been developed in anesthetic pharmacology to enhance anesthesia management, particularly for patients with CVD. Optimal cardiovascular stability, fewer side effects, and improved surgical recovery are the goals of these medications.

One of the most intricate branches of medicine is anesthesiology. The field’s technological and pharmacological advancements have allowed us to execute longer, more complicated surgical operations on more vulnerable patients and, overall, enhance patient outcomes throughout its history. This has resulted in dramatic alterations and radical breakthroughs. However, there have always been risks and difficulties in the field of anesthesiology. Despite progress, it continues to confront the dangers of anesthesia for both patients and doctors, as well as some of the unique difficulties faced by nations with poor or medium incomes. Given the circumstances, it is essential to work together on specific tasks and projects. Furthermore, cutting-edge advancements and technology like robots, genomics, AI, and simulation show potential for enhancing anesthesiology’s patient safety measures and resolving current obstacles, paving the way for safer, more effective, and tailored anesthesia. In order to fully profit from the new technology, however, it is necessary to closely monitor the ethical elements and the trustworthiness of research (Harfaoui et al., 2024). The main goal of new and developing drug innovations is to enhance the pharmacodynamic, pharmacokinetic, and side effect aspects of already existing medications or therapeutic classes by changing their chemical structures. New drugs and methods have emerged in the field of anesthetic pharmacology that seek to enhance anesthesia management, particularly for patients suffering from CVD. These medications are designed to improve cardiovascular stability, decrease adverse effects, and promote a speedier recovery after surgery (Mahmoud and Mason, 2018) (Figure 3).

### 4.1 Cleviprex (clevidipine) in patients with cardiovascular disease

BP is reduced by lowering arteriolar resistance without impacting venous capacitance vessels using the ultrashort-acting dihydropyridine calcium channel antagonist clevidipine (Cleviprex^®^). The drug has a quick start and offset of action. Patients experiencing hypertension before and after cardiac surgery were successfully treated with intravenous clevidipine in the randomized, multicenter, double-blind phase III ESCAPE-1 and ESCAPE-2 studies. Patients undergoing cardiac surgery benefited more from clevidipine than from nitroglycerin or sodium nitroprusside perioperatively and from nicardipine postoperatively in maintaining target range systolic BP, according to the randomized, open-label, multicenter, phase III ECLIPSE trials. Researchers have shown patients having coronary artery bypass graft surgery to have comparable effectiveness to sodium nitroprusside after surgery, and perioperative clevidipine was found to be non-inferior to nitroglycerin in small, double-blind studies. Patients with acute neurological injuries (such as intracerebral hemorrhage, subarachnoid hemorrhage, and acute ischemic stroke) were shown to benefit from clevidipine’s quick BP management, according to non-comparative research (Keating, 2014). Clevidipine enhances cardiac output when peripheral vascular resistance decreases and selectively dilates the arterioles to reduce BP without affecting venous capacitance. Since it has no detrimental inotropic or chronotropic effects, it may be helpful in hypertensive acute heart failure (Peacock et al., 2014). The majority of patients experienced no adverse effects with intravenous clevidipine, and those who did often had little or no reflex tachycardia at all. Researchers suggested that the use of intravenous clevidipine is a very effective method for controlling BP throughout perioperative and ICU periods (Keating, 2014).

Researchers evaluated the relative merits of clevidipine and nicardipine in alleviating perioperative acute hypertension in individuals having heart surgery. Patients who were given clevidipine or nicardipine were included in their retrospective investigation that was conducted at a single center. Individuals were monitored for as long as the medication infusion lasted in the trial, up to a maximum of 48 h. The number of hypertensive episodes per patient, the percentage of time spent within the patient’s specified target BP, the cost of drug therapy, and safety outcomes were all considered results. Researchers indicated that with a little increase in price and no discernible difference in safety, clevidipine improved perioperative BP management compared to nicardipine for patients having heart surgery (Colomy and Reinaker, 2023).

### 4.2 Sugammadex in patients with cardiovascular disease

The steroidal NMBAs rocuronium and vecuronium may be reversed with the application of sugammadex (SG), a new fast-acting drug. As the first non-competitive antagonist for the reversal of NMB, it was licensed for use in adults by the United States Food and Drug Administration (FDA) in December 2015. Most side effects in preclinical studies were mild and resolved on their own. Still, some participants had severe bradycardia and, in rare cases, cardiac arrest within minutes of taking the medication. The causal association between SG and bradycardia is still up for debate since no conclusive mechanism has been suggested despite concerns about its likelihood of bradycardia and anecdotal reports of occurrence (Arends et al., 2020).

Recuperation quality is impacted by postoperative cognitive impairment, which is especially problematic for the elderly and places a strain on healthcare systems. Researchers believed that SG may have a neuroprotective effect that would improve postoperative cognitive function and overall recovery. Researchers assessed the outcomes for patients having cardiac surgery using the ERACS approach: those treated with SG (n = 14) and those treated with neostigmine (n = 7). To assess patients’ cognitive function and overall recovery, the Postoperative Quality Recovery Scale (PQRS) was administered at several intervals after surgery. Cognitive domains showed a favorable postoperative recovery in patients following aortic valve replacement by CPB and the ERACS technique, especially 30 days after surgery when given SG. However, there was no short-term reduction in the duration of ICU and hospital stay. The survey results showed that people really value the restorative characteristics of SG. By inducing the production of anti-inflammatory microglial markers, SG reverses memory impairment that occurs after surgery. Patients having an aortic valve replacement operation using the ERACS technique showed an improvement in postoperative cognitive function after receiving SG therapy. Experimental data from an animal model of minor surgery have confirmed the cognitive impact of SG. Researchers also suggested that SG may have an underlying mechanism, including an influence on glial cells (Muedra et al., 2022).

By incorporating new modalities in surgery, anesthesia, and nutrition, mandating early mobility and feeding, and reducing the surgical stress response, the crucial pathway is expanded, with fast-track surgery serving as an extension of it. Its goal is to cure the patient’s disease while causing them as little physiological harm as possible (Nanavati and Prabhakar, 2014). Researchers examined the effectiveness and safety of SG for fast-track surgery in pediatric patients having heart procedures. Group S children were given 4 mg/kg of SG to reverse neuromuscular block, whereas group N children were given 30 μg/kg of neostigmine and 15 μg/kg of atropine. When used before cardiac surgery, SG may help children have fewer complications, including a shorter extubation time and less postoperative atelectasis. Researchers have proposed SG for fast-track surgery in pediatric cardiac surgery (Li et al., 2021).

Under the influence of general anesthesia, a 68-year-old man had elective surgery to fix a hernia in his abdominal wall. In order to relax the muscles before the procedure, researchers utilized rocuronium. The patient was given 180 mg of SG intravenously once the procedure was finished. The patient had severe bradycardia and hypotension shortly after that; ephedrine was ineffective in treating these conditions. A cardiac arrest without a pulse occurred as a result of this. The patient returned to spontaneous circulation after 4 min of cardiac resuscitation. After a period of stable hemodynamics during which general anesthesia was maintained, the patient emerged from anesthesia without complications. Until his release, his hemodynamic status remained stable. Apart from minor respiratory acidosis and slightly raised D-dimers, post-resuscitation studies, such as the blood tryptase level, were unremarkable. The mechanism of SG-induced bradycardia is yet unclear, although it has been documented before. The research team has concluded that SG caused this instance of cardiac arrest due to the following factors: the sequence of events, the development of pulseless electric activity, and the relatively uneventful clinical history after the arrest. Researchers hope that more people will learn about this possible side effect of a medication that is already widely used (Pereira et al., 2023).

One new drug that may be used to counteract the effects of NMB while under general anesthesia is sugammadex. Some individuals have expressed worry about possible cardiac adverse events (CAEs) based on recent case reports. Out of the nineteen CAEs that were found, two groups were formed: cardiac arrhythmias and coronary artery diseases. A total of 202 patients had bradycardia, 119 had cardiac arrests, 30 had tachycardia, and 22 cases of Kounis syndrome were reported, making these the most common adverse cardiac events. Consistent results were obtained when subgroups were analyzed according to weight, sex, and age. Pulseless electrical activity and cardiac arrest were the most probable CAEs to cause serious complications. There were 51 cases of hypotension, 46 cases of anaphylactic responses, and 23 cases of anaphylactic shock that co-occurred with CAEs. The need for meticulous monitoring and individualized risk assessment is underscored by the fact that researchers have proposed a possible association between SG and CAEs, particularly in individuals who already have cardiovascular risk factors (Lin et al., 2024).

### 4.3 Landiolol in patients with cardiovascular disease

In the perioperative and critical care settings, the fast and short-term management of tachyarrhythmias may be achieved with the approval of intravenous landiolol [Rapibloc^®^ (EU)], an ultra-short-acting, highly cardioselective β1-blocker. Japan has a long history of using it to treat tachyarrhythmias that occur after surgery. Numerous randomized controlled clinical studies have shown that landiolol is effective. Patients with AF/flutter and left ventricular dysfunction and those with postoperative or intraoperative supraventricular tachycardia had their HRs dramatically lowered by landiolol compared to digoxin or placebo. It restored normal sinus rhythm to patients with POAF more effectively than diltiazem. Compared to diltiazem, placebo, or no landiolol medication, the incidence of POAF in the first week after cardiac and other procedures was significantly reduced by perioperative prophylactic injection of landiolol. Moreover, researchers indicated that landiolol reduced the severity of adverse hemodynamics and different reactions to invasive procedures, including electroconvulsive treatment, tracheal intubation, extubation, and percutaneous coronary intervention. The risk of hypotension and bradycardia was minimal with landiolol, and it was well-tolerated in most cases. Compared to esmolol, a short-acting β-blocker often prescribed for the quick regulation of HR, landiolol has better pharmacological characteristics. Present data suggest that landiolol is an effective alternative for the fast, short-term management of tachyarrhythmias; nevertheless, further comparative studies are needed to determine landiolol’s position compared to esmolol. Landiolol comes in two convenient forms: a concentrated liquid and a powder, and it has an easy-to-follow dosing schedule (Syed, 2018).

Researchers assessed how landiolol affected the heart’s reaction to tracheal extubation and waking up from anesthesia. There were 59 patients scheduled for tympanoplasty who did not have any cardiovascular disorders. They were randomly assigned to one of four groups: L1, which received a loading dose of landiolol at 0.125 mg·kg^−1^ min^−1^, followed by an infusion at 0.01 mg·kg^−1^ min^−1^; L2, which received 0.02 mg·kg^−1^ min^−1^; L3, which received 0.03 mg·kg^−1^ min^−1^; and L4, which received 0.04 mg·kg^−1^ min^−1^. Sevoflurane and nitrous oxide were withdrawn from the patient after the procedure, and landiolol was administered instead. Researchers examined the four groups’ MAP, HR, and rate pressure product (RPP) before anesthesia induction, immediately after extubation, 5 minutes after extubation, 10 minutes after extubation, and upon release from the operating room. Compared to the baseline, all groups’ MAPs increased considerably immediately after extubation; all groups’ HRs increased, with the exception of group L4; and all groups’ RPPs increased. Continuous injection of landiolol at doses of 0.03 or 0.04 mg·kg^−1^ min^−1^ may help prevent the increases in HR and RPP that occur during awakening from anesthesia and tracheal extubation, respectively (Shirasaka et al., 2008).

The novel ultra-short-acting β1 selective antagonist, landiolol, has excellent potential in a variety of medical settings, including cardiac and non-cardiac procedures, the prevention and treatment of AF, and the coexistence of heart failure and sepsis. Its ultra-short-acting nature has some benefits in specific contexts, but any adverse effects must be carefully considered. Further studies are required to better understand its function in avoiding AF and its anti-inflammatory actions. Despite obstacles, landiolol shows promise as a therapeutic strategy for the treatment of certain cardiac disorders (Floria et al., 2024).

The prognosis is bleak for patients experiencing an out-of-hospital cardiac arrest (OHCA) due to refractory ventricular fibrillation (VF). To enhance survival rates, it may be possible to overcome refractory VF by selectively blocking beta-1 receptors. Prehospital landiolol’s effectiveness and safety in OHCA and refractory VF are being studied in this experiment. A bolus infusion of 20 mg of landiolol was administered. The duration it took for the trial medicine to induce a sustained return of spontaneous circulation (ROSC) was the leading indicator of effectiveness. Bradycardia and asystole were among the adverse events that occurred. Researchers showed there was no difference between placebo and add-on bolus infusion of 20 mg landiolol in the amount of time it took for patients with OHCA and refractory VF who had already received epinephrine and amiodarone to reach sustained ROSC. Bradycardia and asystole may occur after using landiolol (Gelbenegger et al., 2024).

### 4.4 Dexmedetomidine in patients with cardiovascular disease

An effective non-opioid pain reliever and sedative in the ICU and operating room is DEX. The medicine works as intended, but it is essential to keep an eye on vital signs while using it. The correct dosage must be administered in order to avoid side effects, and the pharmacist and the doctor are jointly responsible for this. Both cardiac and major non-cardiac procedures may benefit from DEX’s potential to decrease POD. The prevalence of delirium is much higher following cardiac surgery compared to non-cardiac surgeries. This might be due to neuroinflammation triggered by the stress of the procedure and the presence of CNS blood products (CNB). As a result, investigating the effects of DEX on the risk of delirium after cardiac surgery with CPB is crucial. Adult patients having cardiac surgery with CPB may have a lower risk of POD if they are given DEX during the operation. On the other hand, the studies show a fair amount of variation. Furthermore, there are a lot of preliminary small-sample trials in the included research, which might cause the positive benefits to be exaggerated. To further validate the potential advantages of DEX in cardiac surgery with CPB, large-scale randomized controlled trials (RCTs) must be designed (Zhuang et al., 2024).

When it comes to noninvasive ventilation (NIV) intolerance, there is no precise ideal sedative regimen. In patients undergoing cardiac surgery who have moderate to severe sensitivity to NIV, this research sought to compare the safety and effectiveness of remifentanil (REM) with that of DEX. There were a total of 179 participants; 90 were given DEX, and 89 were given REM. Generalized estimating equations demonstrated a statistically significant difference after accounting for time, and REM performed better than DEX. Nine patients in the DEX group had adverse effects, three of which were bradycardia and six of which were severe hypotension; no such cases were observed in the REM group. The two groups showed no difference in secondary outcomes such as in-hospital mortality, tracheostomy, ICU LOS, and NIV failure. When looking at the proportion of patients who were able to alleviate their moderate to severe NIV intolerance after heart surgery, this research found no statistically significant difference between REM and DEX. Researchers indicated that after taking time into account, REM clearly outperformed DEX (Hao et al., 2024).

For many years, surgeons have relied on intraoperative opioids to lessen adverse reactions to nociception. Several side effects, some of which may be life-threatening, are associated with opioids. Acute respiratory failure, postoperative cognitive impairment, postoperative ileus (POI), and mortality are among the postoperative problems that patients undergoing cardiac surgery are at high risk of experiencing, many of which are often associated with opioids. These side effects might be lessened using an OFA technique that uses DEX and lidocaine. There have been no published randomized studies in the field of cardiac surgery about this matter. Researchers postulated that significant opioid-related problems after cardiac surgery could be less common if OFA was used instead of opioid-based anesthesia (OBA). Two hundred sixty-eight patients slated for CABG with or without aortic valve replacement were included in this randomized, parallel, and single-blinded clinical study conducted in four French cardiac surgery centers. Patients will be randomly assigned to either an OFA protocol using DEX/lidocaine or a control OBA regimen utilizing remifentanil. When any one of these things happens, that is the primary composite endpoint: 1) postoperative confusion disorder as determined by the Confusion Assessment Method for the ICU test; 2) postoperative infection; 3) acute respiratory distress; or 4) mortality during the first 2 days after surgery. Researchers indicated that pain after surgery, morphine usage, vomiting and nausea, shock, acute renal damage, atrioventricular block, pneumonia, and hospital stay duration are secondary outcomes (Besnier et al., 2024).

As a supplement to anesthesia, ERAS programs use DEX, an alpha-2 agonist. A benefit is that it may help with early extubation and recovery by saving the patient from opioids. Intraoperative infusion of DEX helped the “fast-track anesthesia” when researchers were already utilizing the ERAS platform in 2017, the year the ERAS Cardiac Society was established. The researchers aimed to document their experience and study the possible effects of intraoperative DEX administration in elective heart surgery on patient outcomes as part of the ERAS initiative. The researchers’ studies included 327 patients who had elective cardiac surgery with CPB between 1 June 2017, and 31 August 2018. All patients had the same fast-track anesthetic regimen, which included a parasternal nerve block, decreased opioid dosage, and a DEX infusion, regardless of the type of operation. The main result was the time it took for extubation to be possible after the operation. Three categories were detected: group 0, which included patients who were extubated in the operating room; group <6, which included patients who were extubated in less than 6 h; and group >6, which included patients who were extubated in more than 6 h. Hospitalization expenses, duration of time spent in the ICU, overall hospital stay, and adverse occurrences were the secondary outcomes. According to the research, DEX had no severe side effects and was well-tolerated. One hundred seventy-seven individuals (57%) had their tubes removed early. When comparing the net data from the three groups, Group 3’s LOS in the ICU (median: 70 h vs. 25 h) and overall hospitalization expenses (CHF 62,551 vs. 38,433) were much more significant. With the possibility of early extubation, shorter ICU and hospital stays, and decreased hospitalization expenses, investigators proposed that DEX may be safely utilized as a part of the opioid-sparing anesthetic strategy in patients having elective heart surgery with CPB (Kerroum et al., 2024).

### 4.5 Oliceridine in patients with cardiovascular disease

On 8 August 2020, FDA authorized oliceridine (Olinvyk^®^ Trevena, PA, USA) for clinical use. For such a novel opioid, off-label uses are sure to proliferate, despite the fact that its approved use is considerably limited to alleviate moderate to severe acute pain in adults. Assumed as the “opioid of the century,” its goal is to remove some of the most insurmountable obstacles to prescription opioids, such as the risk of addiction, respiratory depression, and GI side effects, among many others. The novel opioid does this via a whole new mode of action. To reduce the adverse opioid side effects linked to µ-opioid receptors, it focuses on the G-protein sub-pathway rather than the beta-arrestin, all the while maintaining its analgesic properties. However, whether these findings from the phase 2 and 3 studies hold up in actual large-scale clinical usage, both in the US and internationally, remains uncertain. A lot of new ideas in anesthesia have great potential while they are in the laboratory, but when they reach the hands of physicians, they do not hold much water. Some examples are rapacuronium and Althesin (Goudra, 2022).

Oliceridine, a μ-opioid receptor-biased ligand, has just been authorized for the treatment of acute pain in adults who need an intravenous opioid but have not found relief with other methods. Oliceridine produced two peaks in placebo- and baseline-corrected QT (QTc) prolongation in a comprehensive QT (TQT) research that aimed to evaluate its QT liability. The first peak occurred at 2.5 min after a supratherapeutic dosage of 6 mg, and the second peak occurred 60 min after the injection. Finding out how oliceridine extends the QTc interval after a delay in action was the driving force for researchers’ studies. Investigators found that when drugs are taken in and stored within cells, their effects on repolarization are delayed. This makes it difficult to determine whether a drug is prolonging the QT interval or causing arrhythmias when studied acutely. Oliceridine has a minimal risk of developing torsades de pointes due to its multi-ion channel effects, namely, the suppression of late sodium channel current (Burashnikov et al., 2021).

Oliceridine is an innovative ligand μ-opioid receptor agonist that is directed toward G proteins. Injectable oliceridine fumarate at single ascending dosages was the subject of this pharmacokinetic and safety profile evaluation in Chinese patients suffering from chronic non-cancer pain. The safety and pharmacokinetic (PK) studies comprised 32 subjects. Injecting 0.75, 1.5, or 3 mg of oliceridine fumarate intravenously over 2 min resulted in C_max_ values ranging from 51.293 to 81.914 ng/mL and T_max_ values ranging from 0.034 to 0.083 h. Dosage-dependent increases in AUC_0-t_ and half-life (t_1/2_) occurred between 1.85 and 2.084 h. The most often reported side effects of opioids, both during and after delivery, as well as those recorded in the first American studies, were determined to be compatible with treatment-emergent adverse events (TEAEs). Notably, there were no significant side effects noted by researchers. Consistent with the PK findings shown in the first studies performed in the US, oliceridine showed similar PK characteristics and a PK profile in the Chinese population. Doses of 0.75 mg–3.0 mg of oliceridine were well-tolerated and safe for use in Chinese patients suffering from chronic non-cancer pain (Ni et al., 2024).

### 4.6 Remimazolam in patients with cardiovascular disease

Researchers demonstrated that remimazolam, like other benzodiazepines, works by interacting with GABAA receptors; it takes 1–3 min for the drug to start working. The safety and effectiveness of remimazolam for the induction and maintenance of general anesthesia in the non-cardiac surgical population have been investigated in many early randomized controlled studies and meta-analyses. Remimazolam may be beneficial for cardiac anesthesia, according to emerging results from some trials, although the FDA has not yet authorized it for this application. Because of its shorter half-life than that of more conventional medicines, it may help cardiac patients avoid dangerous hemodynamic instability and have a more gradual induction and emergence (Muncan and Bennett-Guerrero, 2024). Inactive metabolites of remimazolam are produced by hepatic metabolism, mainly by esterase enzymes. When esterases in the body break the ester bonds in remimazolam, the inactive metabolite CNS 7054 is formed. The endogenous molecules, such as glucuronic acid, may be conjugated with the metabolites that are produced during ester hydrolysis. In addition to aiding in the drug’s removal, this conjugation makes it easier for metabolites to be excreted by the kidneys. Patients may be quickly roused from anesthesia and drowsiness since remimazolam is rapidly cleared from the body into these inactive metabolites. The fast beginning of action is a significant characteristic that sets remimazolam apart. It is far quicker than many other anesthetics in reaching peak plasma concentrations after intravenous injection; this takes place within minutes (Zhang et al., 2024b).

In 2020, Japan began extensively using the new benzodiazepine remimazolam for general anesthesia. Patients prone to unstable hemodynamics during general anesthesia, such as those with cardiovascular problems, may safely use remimazolam, according to early clinical studies for general anesthesia, as it has fewer cardiodepressant effects than propofol. Coronary angioplasty risks include POCD. Estimates put the rate of PODS following heart surgery at 26%–52%, much higher than the rate after non-cardiac surgeries. The symptoms of POD include reversible cognitive impairment, inattention, changed awareness level, memory, orientation, and sensory abnormalities. A prolonged hospital stay, worse functional recovery, and cognitive impairment in the long run are all symptoms of POD. After heart surgery with CPB grafting, the majority of patients are put under anesthesia to ensure stable hemodynamics and respiratory conditions. Thus, in cardiovascular surgery, including CPB, the therapeutic value of a quicker recovery via sedative antagonistic effects may be debatable. Due to the high prevalence of structural heart diseases in the elderly, such as advanced aortic stenosis and mitral valve regurgitation, and their susceptibility to neurocognitive decline, less invasive cardiovascular surgeries, such as TAVI and MitraClip for mitral valve regurgitation, necessitate early recovery and neurological evaluation. Remimazolam and flumazenil might be used to avoid POD after MitraClip and transcatheter aortic valve implantation (TAVI) (Hirata, 2023).

In order to implant MitraClip^®^ devices in a patient with advanced heart failure and severe mitral regurgitation, researchers used remimazolam as an anesthetic. Remimazolam had less of an impact on the cardiovascular system when used for both the induction and maintenance phases of anesthesia. The patient had a comfortable recovery from anesthesia once the surgical operations were finished, and flumazenil was administered shortly after the trachea was extubated. For individuals with complex and severe CVDs, remimazolam may be the key to effective anesthetic control (Satoh et al., 2021).

During cardiac ablation for AF under general anesthesia, Nam and colleagues examined the effects of remimazolam and desflurane on hemodynamic changes and dosages of vasoactive drugs. Anesthesia was induced with 6 mg/kg/h remimazolam and maintained with 1–2 mg/kg/h in the remimazolam group (n = 78). After propensity score matching, 78 patients were included in the desflurane group. In this group, anesthesia was induced with 1–2 mg/kg propofol and maintained with 6%–10% desflurane. The researchers discovered that the desflurane group had a considerably higher rate of vasoactive drug usage (73% vs. 41%). In addition, the remimazolam group had a substantially decreased incidence rate, duration, and maximum dosage of continuous vasopressor infusion. Remimazolam may have less of an impact on hemodynamic alterations than volatile anesthetics, according to that retrospective research, which included limitations such as a lack of definitions of intraoperative hypotension and a methodology for such changes throughout surgery (Nam et al., 2023). Further research is required to find out whether a volatile anesthetic with cardioprotective properties or a remimazolam anesthetic with reduced cardiodepressant effects during surgery improves outcomes after heart surgery. Researchers showed that when flumazenil is used to counteract them, it leads to fewer postoperative complications (Hirata, 2023).

Remimazolam benzenesulfonate is an alternative to the potentially addictive propofol injections, which is a similar anesthetic that does not need cell P450 enzyme processing and has a shorter elimination half-life. Remimazolam benzenesulfonate outperforms remimazolam in several respects, including its rate of metabolism, the inactivity of its byproducts, and the strength of its medication interactions. So, for diagnostic and surgical sedation, remimazolam benzenesulfonate is a safe and effective option. Remimazolam benzenesulfonate was shown to be more beneficial than propofol in minimizing hemodynamic changes during heart surgery under general anesthesia. Scientists revealed that remimazolam benzenesulfonate decreased the adverse effects of anesthesia by influencing respiratory function and surgical stress response (Tang et al., 2021).

Compared to propofol, the circulatory depressive effects of remimazolam are fewer in individuals undergoing non-cardiac surgery. Remimazolam is not effective or safe for use in cardiac surgery, including CPB. The researchers provided an instance of remimazolam-based anesthetic management during heart surgery with CPB. A left atrial appendage closure, mitral valve repair, tricuspid annuloplasty, and maze operation were all on the agenda for a 76-year-old female patient. During the administration of general anesthesia, researchers administered remimazolam at doses of 6.0 mg/kg/h and 0.6–1.0 mg/kg/h, respectively. The bispectral index value remained within the 36 to 48 range throughout CPB. All vital signs, including BP, HR, and regional cerebral oxygen saturation on both sides, remained within normal limits. Throughout the perioperative phase, remimazolam was not connected with significant problems or intraoperative awareness/recall. Investigators indicated that remimazolam, like other anesthetics now available, may be administered intracardially during cardiac surgery using CPB (Saito et al., 2021).

Researchers demonstrated that remimazolam provides anesthesia with a rate of perioperative adverse events equivalent to other drugs while reducing the requirement for vasopressors and avoiding substantial hemodynamic instability. These results pertain to the induction and maintenance of general anesthesia for catheterization laboratory operations and elective heart surgery. With its favorable hemodynamic and safety profile, remimazolam shows promise for cardiac anesthesiologists in both the operating room and catheterization laboratories. However, additional study is necessary to understand its function in cardiovascular anesthesia completely (Ripoll et al., 2024).

### 4.7 P2Y_12_ inhibitors (cangrelor) in patients with cardiovascular disease

Patients undergoing heart surgery run the risk of potentially fatal complications related to heparin-induced thrombocytopenia (HIT). Two methods exist to avoid intraoperative aggregation in bypass surgery when anti-PF4/heparin antibodies are present: first, employing a different anticoagulant, and second, combining heparin with an antiaggregant. Several publications have reported the practical use of the novel P2Y_12_ inhibitor cangrelor, making it an exciting choice for the latter technique. Researchers conducted an *in vitro* investigation to test cangrelor’s ability to inhibit anti-PF4/Hep antibodies (Abs)-induced platelet aggregation. The study involved combining platelet-rich plasma (PRP) from thirty patients with effective anti-PF4/Hep Abs and five healthy donors. The study examined platelet aggregation in plasma samples spiked with different concentrations of heparin (0.5 IUmL^−1^), normal saline (control), and a combination of cangrelor 500 ng·mL^−1^ and heparin 0.5 IUmL^−1^ (treatment) using light transmission aggregometry. When comparing groups, the Friedman test was utilized, along with the *post hoc* Dunn–Bonferroni test. Investigators showed that cangrelor did not effectively suppress heparin-induced aggregation when anti-PF4/Hep Abs was present. Scientists have determined that cardiac patients impacted by HIT during surgery should not be given cangrelor as a routine antiaggregant. It is recommended to use other tactics until a presurgery aggregation test has shown cangrelor’s effectiveness in a specific patient (Scala et al., 2020).

Maintaining stable circulatory parameters and increasing the patient’s ischemic tolerance are common goals of extracorporeal circulation (ECC) and hypothermia during heart surgery. However, hypothermia and ECC activate platelets, which may lead to malfunction and, ultimately, a fatal coagulopathy. P2Y_12_, the adenosinediphosphate (ADP) receptor, is essential for platelet activation. As a means of protecting platelets against ECC, this experimental investigation examined P2Y_12_ receptor inhibition. Using an *ex vivo* ECC model at normothermia (37°C) and hypothermia (28°C), human blood was exposed to either the short-acting P2Y_12_ blocker cangrelor (1 μM, t_1/2_ < 5 min) or the P2Y_12_ inhibitor 2-MeSAMP (100 µM). Platelet activation and coagulation indicators (thrombin–anti-thrombin complex production) were examined both before and after circulation. Researchers assessed the impact of reversible P2Y_12_ blockage on platelet function in pigs undergoing hypothermic ECC using cangrelor infusion at a rate of 0.075 μg kg^−1^ min^−1^. During ECC and hypothermia, cangrelor blocks P2Y_12_, which limits platelet activation. Platelet inhibition is well-controlled and has a short half-life, which means it can reduce bleeding consequences. Researchers suggested that hypothermia and ECC are two conditions that could benefit from this new pharmaceutical approach (Krajewski et al., 2012).

Since the early use of opioids in the 1930s, the treatment of chest pain associated with AMI has been mostly similar despite improvements in medical and interventional care of the condition. An early study found hemodynamic improvements with opioid therapy, which may explain in part this predominance. It is concerning because opioids slow the gastrointestinal absorption of P2Y_12_ inhibitors, which, in turn, reduces their antiplatelet efficacy. Because there is no evidence from randomized clinical trials to support the use of opioids widely, researchers should rethink how we handle analgesia after a myocardial infarction. AMI chest discomfort is associated with an inadequate delivery of oxygen to the heart muscle. Atherosclerotic plaque and thrombus at the location of plaque rupture usually clog the coronary arteries to a large or entire extent, reducing the oxygen flow. Similarly, worries that morphine reduces the efficacy of antiplatelet medicines have put the long-established first-line analgesic drug under review. Its usage for chest pain was first documented in the 1930s. This medication interaction occurs because opioids cause a delay in the absorption of P2Y_12_ inhibitors in the gastrointestinal tract (GI). Since antiplatelet medications are well-established as necessary in the medical and interventional treatment of AMI, this may have significant ramifications (Fernando et al., 2021) (Table 2).

**TABLE 2:** Novel drugs aimed at improving anesthesia management, specifically in patients with cardiovascular disease.

Anesthesia drug	Mechanism	Function in the cardiovascular system	Advantages in cardiac patients	Reference
Cleviprex (Clevidipine)	An ultrashort-acting third-generation dihydropyridine, clevidipine butyrate preferentially reduces peripheral vascular resistance by blocking calcium influx in arterial smooth muscles, thus exhibiting selectivity for arteriolar vasodilatation	The third-generation intravenous dihydropyridine calcium channel blocker clevidipine has a very short half-life and reduces arterial blood pressure (BP) quickly, to a titratable degree; its effects are eliminated rapidly from the body as a result of metabolism by tissue and blood esterases. Clevidipine, an arterial-selective vasodilator, removes the need to widen the venous capacitance bed to lower peripheral vascular resistance	In patients slated for cardiac surgery, clevidipine was well-tolerated and effective in quickly lowering BP to goal levels prior to operation	Levy et al. (2007)
Sugammadex (SG)	The first of a new family of selective relaxant binding agents, SG (ORG 25969) is a novel cyclodextrin that reverses neuromuscular blockade (NMB) with the aminosteroid non-depolarizing muscle relaxants rocuronium and vecuronium. If you have mild or profound NMB, SG may reverse it	More severe cardiovascular side effects, such as hypotension, third-degree atrioventricular block, and persistent bradycardia, have been highlighted in recent case reports. Patients have had a cardiac arrest after the SG injection, which was caused by coronary vasospasm. Both victims of the cardiac arrest had normal coronaries, although one had non-obstructive lesions	Faster and safer recovery is possible, which is especially helpful for cardiovascular patients who could have trouble with chronic muscular weakness or ventilation	Kapoor (2020)
Landiolol	The β1 selective adrenoceptor antagonist landiolol has a very short half-life. The negative chronotropic impact is more substantial, and the effect on BP is less pronounced compared to other β blockers. Landiolol improved hemodynamics in this trial while dramatically lowering the HR.	Landiolol is a very cardioselective, super short-acting beta-blocker with a half-life (t1/2) of 3–4.5 min that regulates the HR in critical care, emergencies, and perioperative settings	This medication is beneficial for people with tachyarrhythmias, such as atrial fibrillation, because it allows for precise regulation of the HR without significantly lowering BP or airflow	Bezati et al. (2023)
Dexmedetomidine	Dexmedetomidine is an effective non-opioid medication for pain management and sedation in the ICU and during surgery. Despite the drug’s efficacy, vital signs must be monitored while using it. The correct dosage must be administered to avoid side effects, a responsibility shared by both the pharmacist and the doctor	The primary adverse effect of dexmedetomidine is hemodynamic changes. These symptoms include high BP, slow HR, and low BP, which result from the activation of α2-receptors before and after synapses. This leads to the narrowing and widening of blood vessels and reflex bradycardia	It has little impact on respiration, stabilizes the circulatory system, and reduces the need for sedatives and opioids. Additionally, it may reduce stress and myocardial oxygen demand	Chen et al. (2022)
Oliceridine	Opioid medicine oliceridine, marketed as Olinvyk, is prescribed to individuals suffering from moderate to severe acute pain. The injection is administered intravenously (IV)	Utilized for the management of pain in individuals with CVD who exhibit sensitivity to conventional opioids	Even at dosages more significant than their antinociceptive effects, oliceridine’s antinociception is robust and maximum	Burashnikov et al. (2021)
Remimazolam	Remimazolam, sold under the brand name Byfavo, induces and sustains procedural sedation for invasive diagnostic or surgical procedures that last 30 min or less	It is used as a sedative for individuals who need quick surgical recovery and cardiovascular stability	With an incidence of perioperative adverse events similar to other drugs, the data demonstrates that remimazolam provides anesthesia without causing considerable hemodynamic instability and with a decreased requirement for vasopressors	Ripoll et al. (2024)
Volatile anesthetic with cardioprotective effects	Volatile anesthetics enhance recovery after ischemia. Heart surgery patients may have less risk of death and cardiac complications due to desflurane’s and sevoflurane’s cardioprotective characteristics, according to a meta-analysis	Medicated to lessen the risk of myocardial injury during cardiac operations, such as CABG	Among the anesthetic medications that lessen the risk of death and morbidity in surgical patients, desflurane and sevoflurane stand out as having the most significant cardioprotective effects in experimental trials	Lin et al. (2021)
P2Y_12_ inhibitors	Compared to oral P2Y_12_ inhibitors, cangrelor, an intravenous (IV) P2Y_12_ receptor inhibitor, has a faster start and end of the pharmacological activity and is very potent	Cangrelor, prasugrel, and ticagrelor are potent P2Y_12_ inhibitors that increase the risk of serious bleeding but drastically reduce the risk of composite severe cardiovascular ischemic events	Patients at risk of thrombotic events may be more effectively managed with rapid and reversible platelet inhibition, as opposed to the long-lasting effects of oral P2Y_12_ inhibitors such as clopidogrel	Occhipinti et al. (2024)
Milrinone derivatives	Over a therapeutic range of 100–300 ng/mL, milrinone—a bipyridine derivative—has sound inotropic and lusitropic effects, as well as peripheral vasodilation and mild chronotropic effects. Decompensated congestive heart failure is a condition that milrinone is used to treat	Milrinone, a bipyridine derivative, mainly inhibits type III phosphodiesterase, preventing the catabolization of cyclic AMP. Its principal cardiovascular effects include the direct stimulation of cardiac contractility and dilatation of arteries and veins	Help high-risk cardiac patients with inotropic assistance without significantly increasing the demand for oxygen by the heart, as is the case with conventional inotropes	Pietrangelo et al. (2010)
Liposomal bupivacaine (LB)	The Food and Drug Administration has authorized the use of LB for transversus abdominis plane (TAP) blocks as part of this expansion. Bupivacaine, in its extended-release liposomal form, may continue to work for up to 3 days after an infiltration	The extended duration of action of multivesicular liposomes containing bupivacaine has led to their greater use. Research has shown that the local infiltration of LB extends the duration of action and delays peak plasma concentration compared to bupivacaine hydrochloride. Because LB lasts longer than conventional bupivacaine, it can more effectively regulate postoperative pain	Patients who are administered LB showed a considerable improvement in their nausea ratings, a reduction in their maximum pain scores across all time points examined, and a marked decrease in their overall opioid use within 72 h after injections	Song et al. (2021); Tong et al. (2014)

## 5 Anesthetic drug delivery optimization in patients with cardiovascular disease

One of the most significant issues in the fields of anesthesiology and pain treatment is LA cardiotoxicity. Using human-induced pluripotent stem cell-derived cardiomyocytes (hiPSC-CMs) as a model, researchers examined the various forms, risk factors, treatment strategies, and mechanisms of LA-induced cardiac damage. Scientists quickly reviewed the documented cardiotoxic effects of several LA medications, including those associated with esters and amides. The researchers’ study also included the following topics: pharmacokinetics, pharmacodynamics, sodium channel dynamics with respect to individual variability and genetic determinants, techniques for avoiding and controlling cardiotoxic effects generated by LA, clinical manifestations of these effects, and cardiotoxicity. The cellular model of hiPSC-CMs was extensively examined, along with its uses and significance, in assessing the cardiotoxic effects of LA medications. Researchers also discussed the possibilities of hiPSC-CMs in risk evaluation, medication screening, and the creation of tailored treatments. Researchers showed that sodium channel disruption, reactive oxygen species (ROS) generation, and immune system response abnormalities caused by LA medications were the primary mechanisms of LA-induced cardiotoxicity. In addition, factors such as pharmacokinetics and pharmacodynamics, which are unique to each medication, have a significant role after LA drug injection. Patients’ unique characteristics—including their age, number of co-morbidities, and genetic variability—highlight the need to tailor risk mitigation and safety measures to each individual. Researchers suggested that careful dosing, constant monitoring, and the quick availability of resuscitation equipment are the three most important aspects of the LA cardiotoxicity prevention and treatment measures discussed (Jiang et al., 2024).

A lot of people have utilized LAs in therapeutic practice because they temporarily reduce pain by blocking the passage of nerve impulses. Neurotoxicity and short half-lives, on the other hand, have severely restricted their use in therapeutic settings. Several DDS have been developed to encapsulate LA drugs, allowing for the delayed release of high dosages and the provision of analgesia over an extended time, thereby circumventing these limitations. Many different types of LA carriers have been studied so far, and some of them are even available for purchase. The most researched of them have been delivery systems based on polymers, particularly polymeric nanoparticle carriers (Wang et al., 2019). DDS is a dynamic, interdisciplinary area that is constantly changing. Due to innovative DDSs and monitoring technology, anesthesiologists will be able to provide safer, easier, and quicker anesthetic procedures in the future (Dave et al., 2017).

Researchers tested a new automated anesthesia system using CPB for closed-loop administration of IV anesthetic medications during cardiac surgical operations. All three parts of general anesthesia—hypnosis, analgesia, and muscular relaxation—are combined in one medication administration robot for anesthesia. Out of the 16 patients who had heart surgery using robotic anesthesia, 80% were successful. Issues with the automated anesthetic administration system caused the exclusion of four patients from the final study. According to the secondary qualitative observations by researchers, hypnosis effectively controlled the pain for 70% of the maintenance time, whereas analgesia only worked for 3% of the maintenance time. For cardiac surgeries requiring CPB, the fully automated closed-loop device that investigators evaluated might be a safe and effective option. The clinical performance of anesthetic control was found to be good in the current experiment (Zaouter et al., 2016).

A wide variety of hydrophilic, hydrophobic, or amphiphilic chemicals may be transported and potentially delivered using lipid-based drug delivery systems (LBDDSs), which are helpful and biocompatible nanocarriers. When compared to free medicines, which may have poor targeting, increased systemic toxicity, and the risk of drug resistance, LBDDSs provide the benefit of tailored drug delivery. Nanoemulsions, microemulsions, lipid vesicles, and lipid nanoparticles are the four main types of lipid carrier systems used to transport pharmaceuticals, each with its own set of advantages and disadvantages in terms of pharmacological characteristics and therapeutic uses. With the second generation of LBDDSs, surface chemistry may be modulated by changing or functionalizing the composition of the lipid layer, marking a significant advancement in the age of tailored drug delivery beyond the superficial standard vesicles. To improve targeted medication delivery and decrease toxicity, lipid carriers have had their surfaces modified with enzymes, peptides, antibodies, and small molecules. Regarding this matter, the first innovative method that enhances blood circulation time and decreases opsonin resistance and clearance was the polyethylene glycosylation (PEGylation) of the lipid surface. A potential new strategy to treat certain tumors and overcome drug resistance is the targeted-liposomal approach, which uses multi-drug and multi-receptor tactics. There are a number of liposomal formulations that are second-generation conventional DDSs that are currently in different phases of clinical testing. In order to improve the DDS further, you could also test ways to increase disease targeting via surface functionalization with other entities (Srivastav et al., 2023).

Researchers summarized all 76 randomized controlled studies that have ever been published on the topic of postoperative pain management with LB (EXPAREL; Pacira Pharmaceuticals, United States). Only four out of thirty-six studies found that LB improved the main result in a clinically meaningful and statistically significant way compared to unencapsulated bupivacaine or ropivacaine when infiltrated surgically. Eleven out of twelve studies, or 92%, found that peripheral nerve blocks using unencapsulated bupivacaine were more effective than infiltrating LB in reducing pain. Out of sixteen experiments testing peripheral nerve blocks with either liposomal or unencapsulated bupivacaine, the results were inconsistent. Statistically significant differences for primary outcome measure(s) were reported by 84% (16 of 19) of trials assessed to have a high risk of bias, compared to 14% (4 of 28) of studies deemed to have a low risk of bias. LB is not superior to regular LAs, according to the available research (Ilfeld et al., 2021).

When injected perineurally, LB is believed to prolong the analgesic effects of peripheral nerve blocks. Perineural LB has conflicting reports on its efficacy in therapeutic settings. Comparing non-liposomal LAs to perineural LB, researchers determined whether one provides better peripheral nerve block analgesia. LB and non-liposomal LAs were evaluated in randomized studies that examined the efficacy of peripheral nerve block analgesics. The main point of the evaluation was the variation in the area under the receiver operating characteristic curve (AUC) of the combined rest pain severity ratings from 24 to 72 h. Patient satisfaction, duration of hospital stay, functional recovery, the incidence of opioid-related side effects, length of time to first analgesic request, intake of postoperative analgesics, and LB side effects were all considered secondary outcomes. Researchers evaluated the AUC pain ratings based on a 2.0 cm·h minimally clinically relevant difference. Researchers also examined nine studies with 619 people. After combining all trials, the AUC pain ratings ±SD from 24 to 72 h for non-LB were 7.6 ± 4.9 cm·h, and for LB, they were 6.6 ± 4.6 cm·h. Hence, when contrasted with non-LB, perineural LB improved the AUC of pain ratings from 24 to 72 h by 1.0 cm·h, which was a clinically insignificant advantage. The difference between the groups was not found to be significant when an industry-sponsored experiment was excluded. In terms of pain severity at individual time points, up to 72 h, analgesic consumption, time to first analgesic request, opioid-related side effects, patient satisfaction, length of hospital stay, and functional recovery, secondary outcome analysis did not find any additional benefits to LB. There were no documented adverse effects of LB. Compared to conventional LAs, perineural LB improved the AUC of postoperative pain ratings in a statistically significant but clinically insignificant way. Excluding an industry-sponsored experiment also made this advantage nonsignificant; following surgery, LB was no different from ordinary LAs in terms of postoperative pain and any other functional and analgesic outcomes. Perineural LB is not superior to non-LB for peripheral nerve blocks, according to high-quality data (Hussain et al., 2021).

It may be challenging to manage postoperative pain after heart surgery, and problems might arise if the discomfort is not well controlled. Because LB lasts longer than conventional bupivacaine, it is better able to regulate postoperative pain. Unfortunately, there is a lack of information on how well it works in congenital cardiac surgeries. Researchers determined whether pediatric patients having cardiac surgery by median sternotomy needed more opioids—LB or plain bupivacaine—for local infiltration. Use of antiemetic medicine, duration of stay, pain ratings, and adjunct pain medication were included as secondary outcomes. In terms of demographics, the two groups were identical. There was no statistically significant difference in opioid usage between the groups given LB or ordinary bupivacaine after surgery. In contrast to the ordinary bupivacaine group, those given LB were more likely to use acetaminophen and antiemetic medications. In terms of secondary outcomes, such as duration of stay, no significant changes were observed. Researchers revealed that it is possible that LB does not provide any benefit over normal bupivacaine for the management of pain after congenital heart surgery (Tanner et al., 2024).

For pecto-serratus/serratus anterior plane blocks, a biphasic pattern was observed with the combination of plain and LB injections; the maximum arterial plasma concentrations were detected within 30 min. Nearly one-third of individuals had maximum values that were higher than the potentially harmful threshold, although no clinical signs of toxicity were observed. Clinicians need to be cautious when using plain and LB together for thoracic fascial plane blocks (Alfirevic et al., 2024).

Longer recovery times, surgical complications, and persistent pain may all result from inadequate pain management after heart surgery. When performing wound infiltration in a median sternotomy, the authors postulated that combining LB with plain bupivacaine (PB) would result in more effective and longer-lasting analgesia. Pain ratings, opioid usage, and adverse events were monitored for 72 h after patients were treated. Postoperative analgesia was not markedly improved when LB was given to PB for sternotomy wound infiltration after elective heart surgery (Subramaniam et al., 2023).

Researchers evaluated the analgesic efficacy of LB compared to plain LAs in a meta-analysis and comprehensive review for people receiving fascial plane blocks in the abdomen. When applied to the fascial plane of the abdomen wall, researchers found that LB and ordinary LAs had comparable analgesic efficacy. According to the investigators, LB is not preferable to normal LAs for abdominal fascial plane blocks (Hussain et al., 2024).

## 6 Conclusion

Contemporary cardiac surgery relies heavily on anesthesiology. It ensures that patients are safe and comfortable during surgical procedures. Intubation, breathing, monitoring the circulatory system, controlling BP, and depth of anesthesia are the main procedures of anesthesiology in cardiac surgery. Managing hemodynamic parameters, monitoring cardiac and respiratory function, maintaining sufficient analgesia, and minimizing consequences, including myocardial ischemia, arrhythmias, hypothermia, and thromboembolic events, are just a few of the many issues that specialists encounter. Ensuring hemodynamic stability during cardiac surgery is a significant challenge for anesthesiologists. As the research progressed, it became clear that the anesthesiologist’s preferences, the patient’s health, the nature of the surgery, and the patient’s condition all had a role in determining the anesthetic method employed. When deciding, it is essential to weigh the benefits and drawbacks of each approach (Novikova et al., 2024).

Recent developments in cardiac anesthesia have revealed information gaps that need a shift in emphasis toward perioperative treatment, particularly with regard to long-term results. Patients will eventually benefit from research that incorporates new types of data, uses unique analytics, and explores innovative research methods. The efficient use of opioids and ultrasound-guided regional anesthesia for pain reduction has facilitated ERACS with the rise of minimally invasive cardiac surgery (MICS). For safe cardiac anesthesia, perioperative imaging, such as 3D TEE, together with novel equipment and drugs, big data analytics, and algorithms produced by machine learning, will be necessary. Some, like leadless pacemakers and MICS, have anesthetic implications; others, like software augmented with AI, improve imaging, which may lead to quicker interpretation of results and better perioperative care. Heart failure mortality and worsening have significantly been reduced because of medical treatments like dilators and diuretics (Ramachandran et al., 2023).

Innovations in technology, such as AI, new medications, regional anesthetic methods, and better imaging, have caused cardiac anesthesia to progress significantly. Improvements in perioperative care, patient safety, and postoperative recovery have resulted from these innovations. Precise preparation, clear communication, and interdisciplinary teamwork are essential for the increased difficulties posed by minimally invasive cardiac procedures. Clinical decision-making and diagnostic enhancement powered by AI provide even more chances to improve patient care. Improving results in the ever-changing field of cardiac anesthesia will need persistent study, the incorporation of new technology, and an emphasis on individualized patient care (Mathis et al., 2024).

The patient’s cardiovascular health, preexisting diseases, and treatment type determine the precise medication selection and dose. In order to regulate medication delivery and maintain patient stability during the process, it is crucial to carefully monitor hemodynamic parameters such as BP and HR. The goal of the most recent developments in cardiac anesthesia has been to decrease risks and increase accuracy in patient outcomes during cardiac surgeries. By lowering the risk of hemodynamic instability, improving recovery, and lessening the severity of side effects, these new medications allow anesthesiologists to provide cardiovascular patients with better, more individualized treatment. To guarantee positive results for patients with cardiovascular conditions, meticulous preparation, close observation, and individualized anesthetic treatment are required. The safety and efficacy of anesthesia in this high-risk group are being further improved by emerging technology and a trend toward tailored and less intrusive techniques. Improvements in patient safety, optimization of outcomes, and faster recovery are driving the future of cardiovascular anesthesia, influenced by developments in personalized medicine, technological integration, and pharmacology. There will be a more significant focus on patient-centered treatment and safety in the future of cardiovascular anesthesia, which is expected to be data-driven, individualized, and minimally intrusive. These developments may improve the efficiency and safety of cardiovascular treatments while simultaneously improving patient comfort and increasing the success rate of these procedures. Anesthetic medication side effects in cardiac patients may be successfully reduced with the use of new pharmaceuticals and novel management strategies, including AI. Each new approach requires advanced and specialized study. The dosing, administration, and treatment of novel cardiac anesthetic medications, as well as their adverse effects, need to be addressed through systematic reviews and meta-analyses. Researchers can also improve the accuracy and effectiveness of anesthetic medications while decreasing their adverse effects by exploring innovative drug delivery strategies, such as employing nanoparticles to administer cardiac anesthetic agents. Thus, novel cardiac anesthetic medications may be significantly advanced through the joint efforts of biotechnologists, pharmacists, anesthesiologists, and cardiologists.
